# The Effects of Daily Life Auditory Demands on Listening Effort, Affect, and Fatigue as a Function of Hearing Loss

**DOI:** 10.1177/23312165251413329

**Published:** 2026-03-03

**Authors:** Nicole A. Huizinga, Laura Keur-Huizinga, Adriana A. Zekveld, Sophia E. Kramer, Eco J.C. de Geus

**Affiliations:** 1 Department of Biological Psychology, Faculty of Behavioral and Movement Sciences, 1190 Vrije University Amsterdam, Amsterdam, the Netherlands; 2 Amsterdam Public Health Research Institute, Amsterdam University Medical Center, Amsterdam, the Netherlands; 3 Department of Otolaryngology-Head and Neck Surgery, Amsterdam University Medical Center, Amsterdam, the Netherlands

**Keywords:** listening effort, fatigue, affect, EMA, daily life, hearing loss, auditory environment, hard of hearing

## Abstract

Previous research has highlighted challenges for individuals with hearing loss, including increased listening effort and fatigue. This study aimed to: (a) examine the relationship between auditory demand and listening effort, affect, and fatigue, focusing on the moderating role of hearing loss; and (b) assess whether listening effort and affect mediate the effect of auditory demand on fatigue. A total of 130 participants, with and without hearing loss, participated in EMA over 5.5 days, answering questions on listening effort, fatigue, and listening environment attributes. Auditory demand was defined by contextual and subjective components derived from EMA responses. LME models analyzed the effect of auditory demand on listening effort, affect, fatigue and the moderating role of hearing loss. Additional models tested mediation by listening effort and affect. Results: highlighted that both contextual and subjective auditory demand significantly increased listening effort with stronger effects in those with more hearing loss. No effects of contextual auditory demand on affective state were observed, nor was there a moderation effect of hearing loss. An effect of subjective auditory demand on affect was observed, but no moderation of hearing loss was present. Contextual and subjective auditory demand predicted fatigue (β = 0.07–0.14, *p < .*01–*p* < .001) with amplified effects present in those with more hearing loss (*p*_interaction_ < .01) for contextual demand. Mediation analyses highlighted that listening effort contributed to the demand-fatigue relationship, though patterns differed by demand type. The results indicate that increased listening effort, rather than negative affect, may underlie the association between auditory demand and fatigue.

## Introduction

As the world's aging population is living longer, the societal burden of hearing loss is growing. The World Health Organization (WHO) projects 2.5 billion people to have some degree of hearing loss by 2050 and that roughly 700 million will need hearing rehabilitation (World Health Organization (WHO), 2021). The complaints of those with hearing loss cover a wide range of physical to psychosocial issues. A few notable complaints include increased listening effort, stress and fatigue. These items are pressing given that they can also lead to reduced quality of life and social isolation, particularly in aging groups (Nordvik et al., 2018).

Until recently, the construct of fatigue in those with hearing loss has received relatively little systematic attention. Fatigue is commonly defined as, “a feeling of weariness, tiredness or lack of energy” (O’Connor, 2004; Tiesinga et al., 1996). In daily life, most people experience fatigue as result of physical or mental demands, and these feelings are usually short-lived, with recovery achieved through rest or breaks. This concept of daily fatigue is normal and does not negatively impact quality of life (Shen et al., 2006). However, there is an important distinction between this construct of everyday fatigue and the more intense, persistent fatigue often reported by those with hearing problems. For these individuals, even routine activities, such as following a conversation at work, can lead to pronounced and sustained fatigue that may negatively impact quality of life (Davis et al., 2021; Hornsby et al., 2016).

One potential mechanism that could affect the fatigue recorded in those with hearing loss is the increased listening effort required to process auditory signals in day-to-day life (Holman et al., 2019). Compared to normal hearing control participants, those with hearing loss experienced elevated levels of listening effort across speech intelligibility levels representative of daily-life conditions in a study by Ohlenforst et al. (2017). Because understanding speech is vital for social functioning, hard of hearing persons can be motivated to exert the required listening effort, even for prolonged periods of time (Holman et al., 2019). Listening effort may therefore have impact on parallel task-related cognitive processing, because the limited available cognitive resources are partially redirected (Hussein et al., 2022). In addition, reduced performance on the ongoing task and/or actual speech perception may give rise to negative affect (Holman & Naylor, 2025). Given that both listening effort and negative affect are known to engage the physiological stress systems, the prolonged state of heightened effort and negative affect, compounded by a heightened physiological arousal state, could jointly increase the risk for fatigue.

Research addressing the intersection of hearing loss and fatigue stems from a small number of qualitative and quantitative studies (Alhanbali et al., 2017; Burke & Naylor, 2020; Dwyer et al., 2019; Holman et al., 2019; Hornsby et al., 2016). Alhanbali et al. (2017) used a fatigue assessment scale (FAS) and an effort assessment scale (EAS) to investigate fatigue and listening effort in a sample of 200 participants with four groups: hearing aid users, cochlear implant users, a group with single-sided deafness and a control group. They found significantly higher scores of fatigue in all of the groups with hearing loss compared to the control group (Alhanbali et al., 2017), but no significant difference between the three hard of hearing groups. Hornsby et al. (2014) used a different fatigue measure, the PedsOL Multidimensional Fatigue Scale, in children with a range of hearing loss. This scale assessed multiple fatigue domains (general, sleep/rest, and cognitive fatigue) which ultimately provided a total fatigue score. Their work showed higher fatigue in children with hearing loss compared to an age-matched control group. Dwyer et al. (2019) used the Profile of Mood States (POMS) to assess general fatigue and developed three additional questions to specifically evaluate listening-related fatigue. These questions targeted the physical and emotional tiredness caused by listening difficulties and addressed how often participants experienced fatigue related to listening. The study highlighted that while POMS did not show significant differences in fatigue between participants with and without hearing loss, the listening-specific fatigue questions revealed significant differences, especially for those with hearing losses, who reported more frequent and severe listening-related fatigue (Dwyer et al., 2019). Using a qualitative approach, Holman et al. (2019) held interviews with hard of hearing participants to better understand their complaints in daily life. They identified two primary sources of fatigue: effort-related and emotion driven (i.e., negative emotions about having hearing loss) (Holman et al., 2019).

Despite the efforts to better understand fatigue in those with hearing loss, few steps have been taken to repeatedly sample and assess fatigue in those with hearing loss outside of the laboratory environment. A clear exception is from the work of Burke and Naylor (2020), whereby researchers conducted an EMA study in 24 participants with and 20 without hearing loss. For two weeks they prompted questions about the listening environment and momentary fatigue but did not find a difference in fatigue patterns in the group with hearing loss compared to the controls (Burke & Naylor, 2020). Similarly, another recent EMA study did not find differences in fatigue between those with normal hearing and those with hearing loss (Jorgensen et al., 2025). Apart from the aforementioned studies, no other attempts have been made to examine the impact of hearing loss on daily fluctuations in fatigue as a consequence of the auditory demand of the subjective, social and environmental context, as well the subjective listening effort in response to those situations. This could be attributed to the complicated nature of collecting such data in daily life settings.

To help fill this gap, and to better understand dynamics of auditory demand, listening effort, affect and fatigue, we opted to repeatedly sample these items in a group of people during their daily lives. All measures of interest (auditory demand, affect, listening effort and fatigue) were derived from an ecological momentary assessment (EMA) procedure across a 5-day period (7 beeps per day) in participants that were selected to show large variation in hearing loss (as assessed by pure tone audiometry). Auditory demand was conceptualized by contextual and subjective components. Contextual auditory demand was denoted by (a) environmental demand (according to the listening situation) which stemmed from the CoSS framework (Wolters et al., 2016) and (b) social demand (according to the social element of who they were with). Subjective auditory demand was based on the Framework for Understanding Effortful Listening (FUEL) (Pichora-Fuller et al., 2016) which outlines that listening effort is a product of auditory task demand, or difficulty level and motivation to perform well on the listening task. Using an EMA question about listening difficulty to reflect difficulty level and an EMA question about importance to hear well to reflect motivation, the product of these two questions was regrouped to a rank score of subjective auditory demand. Affect was defined using a scale to assess “how you feel in the moment,” and listening effort and fatigue were scored on a Likert scale following the approach of a previous ambulatory study in participants with hearing loss (Burke & Naylor, 2020).

The aims of this study were two-fold. First, we assessed the effect of auditory demand (separated by between and within-person components) on listening effort, affect and subjective fatigue with the expectation that the within-person effect will be amplified by greater hearing loss (i.e., moderation of hearing loss). And second, we aimed to assess the potential mediating role of listening effort and affect on the (moderated) effect of auditory demand on fatigue.

Regarding aim 1, we hypothesized that moments of increased auditory demand would be associated with higher levels of listening effort, worsened affect, and higher levels of fatigue and furthermore that these within-person effects would be stronger in participants with more severe hearing loss, reflecting the moderating role of hearing loss. In regard to aim 2, we hypothesized that the within-person effect of auditory demand on fatigue, and its moderation by hearing loss, would be mediated by both increased listening effort and negative affect. Our study seeks to add to the body of research to assess fatigue in the daily life of participants with hearing loss. Currently, little is known about this important corollary of hearing loss, whereas increased understanding of the antecedents of fatigue could offer new ways to monitor and intervene the long-term impact of hearing loss on daily wellbeing and health.

## Methods

### Population

A total of 136 participants (mean age: 57.1 years; females: 96; males: 40) with and without hearing loss were recruited for study participation. All were native Dutch speakers and had normal or corrected-to-normal eyesight. Exclusion criteria included use of psychoactive drugs, use of drugs influencing ANS activity (e.g., Beta-blockers, Anticholinergics), a history of metabolic disorders, serious alcohol abuse, serious heart disease(s), diagnosed neuropathy as well as using a cochlear implant. Participants with hearing loss were recruited via the audiology clinic at the Amsterdam UMC, hearing-related newsletters and social media. Participants with and without hearing loss were recruited via flyers hung around Amsterdam, social media, and via word of mouth. Participants received a payment for study completion in addition to reimbursement for travel costs. All participants provided informed consent before commencing the experiment and the study was approved by the medical ethical committee of Amsterdam UMC, location VUmc.

### Procedure

The duration of study participation was 5.5 days. Participants started the study at the Amsterdam UMC, location VUmc, where they completed informed consent and subsequently underwent a set of tasks, including standard audiometry testing to quantify hearing loss, in the laboratory environment. At the end of the laboratory session participants downloaded an application which prompted surveys to their own smartphone for 5.5 days. The study here reflects one part of a larger scale project, entitled “REAL-HEARING,” which also contains a laboratory component (Keur-Huizinga et al., 2024) and an ambulatory physiological component (Huizinga et al., 2025).

### Hearing Loss Assessment

Participants underwent a standard pure-tone audiometry procedure with a trained researcher, or licensed audiologist in cases with more severe hearing loss. Each participant sat inside a sound booth and wore headphones for the procedure. Air-conduction thresholds, in dB HL, were measured at frequencies of 250, 500, 1000, 2000, 4000 and 8000 Hz for the left and right ear. Participants indicated detection of auditorily presented tones by pressing a button. The Hughson-Westlake procedure was used to estimate the thresholds (Carhart & Jerger, 1959; Hughson & Westlake, 1944). The pure tone average (PTA) was calculated by averaging the thresholds at 500, 1000, 2000 and 4000 Hz of the better ear (Durrant & Lovrinic, 1984).

### EMA

**Figure 1.**
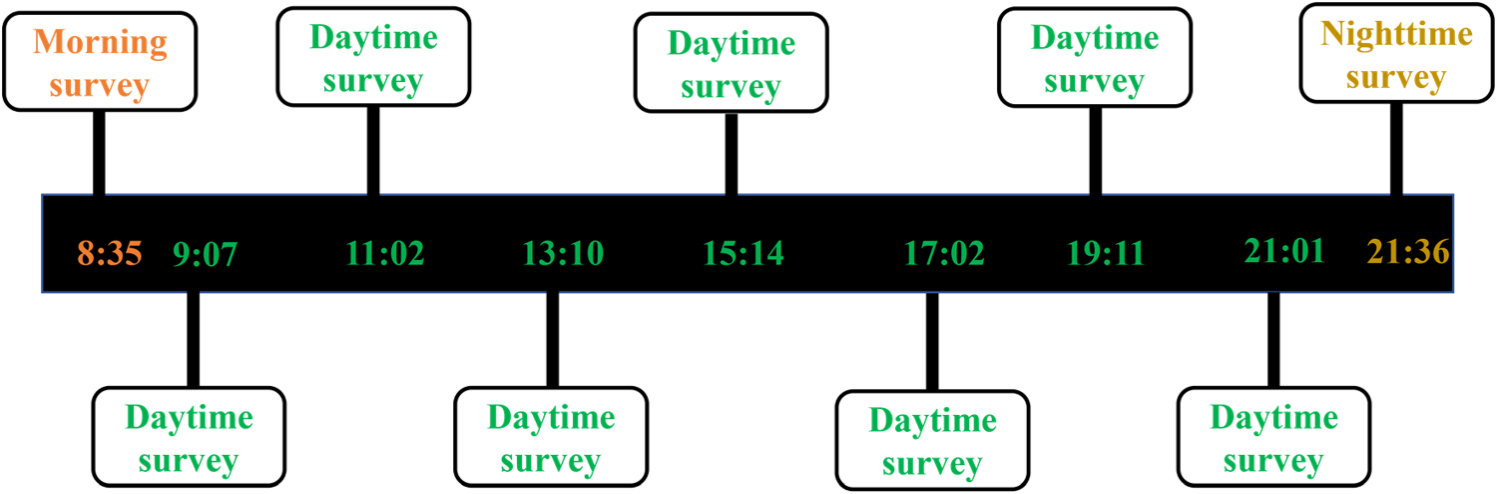
Example EMA prompt schedule on a single day of study participation. Numbers on the timelines highlight example hours of the prompt schedule.

Participants downloaded the Avicenna smartphone application (https://avicennaresearch.com) which prompted surveys 9 times per day (one morning survey, seven daytime surveys and one nighttime survey) for 5.5 days. For a full list of questions, see Supplementary Table 1. The daytime survey consisted of 18 questions about mood, the hearing environment, and fatigue, and were prompted roughly every two hours between 9.00 and 21.30, with each prompt randomly delivered within a 30-min window (e.g., between 9:00–9:30, 11:00–11:30, 13:30–14:00, 15:00–15:30, 17:00–17:30, 19:00–19:30, and 21:00–21:30). The exact timing within each window was determined by a uniform random jitter of up to ±15 min to reduce predictability. Participants were instructed thoroughly on how to use the application and were told to fill in each survey as soon as possible after receiving a notification. Each daytime survey was available for 40 min before expiring and participants received a reminder notification 10 min after the initial prompt. The morning and nighttime surveys were notably shorter (four and five questions, respectively) and asked about sleep quality as well as sleep and awake times. These surveys were prompted between 8.30 and 9.00 (morning) and 21.30 and 22.00 (nighttime) and had no expiry time. An example timeline of a single day of the prompt schedule is shown in Figure 1.

The daytime EMA questions used for this study included queries about listening environment items, affect, listening effort, and fatigue. The questions related to the listening environment included questions about how difficult it was to listen, how important it was to listen, who they were with and the listening situation. All of these questions referred to the past 15 min. The listening difficulty was assessed using an ordinal variable with categorical response options. Participants were asked, “How difficult was it to hear/listen in the past 15 min?” The response options included “Very easy,” “Easy,” “Neither easy nor difficult,” “Difficult,” and “Very difficult.” The variable was treated as ordinal (1‒5) due to the ordered nature of the categories. The importance of hearing in the past 15 min was also assessed using an ordinal variable with categorical response options. Participants were asked, “How important was it to hear well in the past 15 min?” The response options included “Not important,” “Somewhat important,” “Important,” “Very important,” and “Extremely important.” Like the *hearing difficulty* question, this variable was also treated as ordinal (1‒5) due to the ordered nature of the categories. The question “Who were you with in the past 15 min?” was assessed using a categorical variable with seven response options. To help capture the social context, participants were asked to select *who they were with* in the past 15 min, with the following choices: “Alone,” “Partner,” “Children,” “One or more colleagues,” “Family (besides your partner and children),” “Friend(s)” and “Other.” And lastly, the listening situation in the past 15 min was assessed using a categorical variable with seven response options. Participant could choose multiple options from the following: “Conversation with 1 person,” “Conversation with more than 1 person,” “Phone conversation,” “Actively listening to a speaker (live),” “Actively listening to TV, radio, etc.,” “Ambient noise” and “Passive listening, not an active listening task.”

Questions to assess affect were taken from the Maastricht Mood Questionnaire (Myin-Germeys et al., 2001). Participants used a slider scale from 1 (not at all) to 7 (very) to answer whether they felt relaxed, cheerful, enthusiastic, and content. These scores were averaged to denote a positive affect score. The negative affect score was the average score from questions about being insecure, lonely, anxious, irritated and down, also on a slider scale from 1 to 7. To assess overall affect, participants were also asked, “How are you feeling now?,” on a scale from 0 (very bad) to 10 (very good). With regard to listening effort, participants were prompted, “how much effort did it take to listen in listening situations in the last 15 min,” from 0 (no effort) to 10 (extremely high effort). With regard to fatigue, participants were asked to*, “*Rate your fatigue by selecting the number that best describes your fatigue at the moment,” from 0 (no fatigue) to 10 (extreme fatigue)*.*

We note the temporal misalignment between affect/fatigue and effort measures: affect and fatigue were assessed *in the moment*, whereas listening effort referred to the *preceding 15 min*. This approach follows other EMA conventions that capture affect as a momentary state (van der Mee et al., 2022; Stone et al., 2023) and conceptualizing listening effort as a recent-window experience (von Gablenz et al., 2021; Wu et al., 2020)

Additional questions from the morning survey were used to gather information about sleep quality and duration. Participants were asked, “How did you sleep?” on a slider scale from 1 (bad) to 7 (good). Sleep quality was taken as the average across the days of study participation. Participants were also asked, “What time did you go to sleep last night?” and “What time did you wake up this morning?.” Sleep duration was taken as an average of this reported window from two days of study participation and verified by accelerometry data which was available as part of the ambulatory physiological component of the larger project for these two days (Huizinga et al., 2025).

### Analysis Strategy

Some missing data were present due to non-responses to EMA prompts. No specific imputation or handling techniques were applied, and analyses were conducted using available data for each prompt. The core set of analyses, using the available data, involved (a) dimensionally reducing the EMA questions about the auditory demand. We transformed the scores to create a new variable defined as “subjective demand,” which was based on the importance and difficulty ratings. In addition, “contextual demand” included both social demand (who participants were with) and environmental (listening situation) demand. (b) We provided overviews of the correlations between the relevant daytime EMA questions, as well as the dimensionally reduced measures, using between and within-person approaches and c) ran linear mixed models to address our core study aims on the effects of each auditory demand variable, as well as its moderation of PTA, on listening effort, affect and fatigue. Then lastly, we tested the mediation of listening effort and affect in models which tested the (moderated) effect of auditory demand on fatigue. Details are described below.

### Dimensional Reduction of Categorical Questions to Yield Contextual Auditory Demand Measures

To create measures of contextual auditory demand we used the EMA questions regarding the “listening situation” and “who you’re with” to capture the environmental and social context of listening, respectively. Both of these aspects can affect the burden of listening (Lemke & Scherpiet, 2015; Podury et al., 2023).

**Figure 2.**
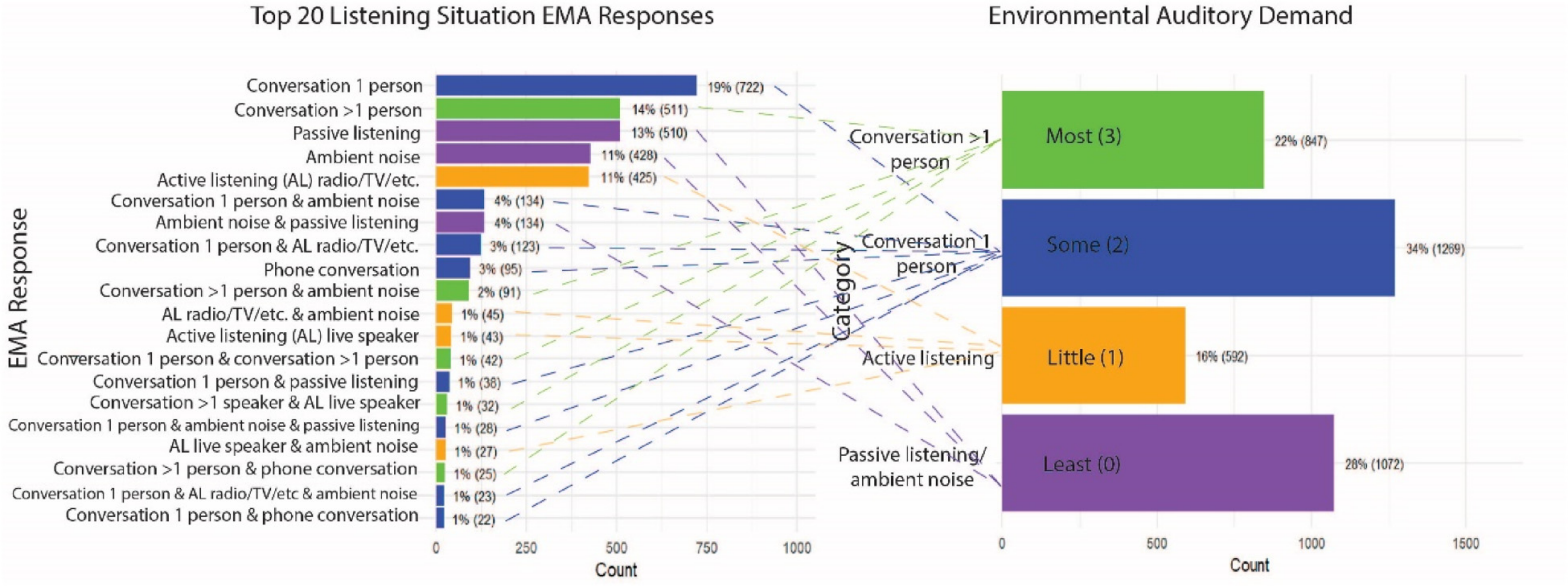
Dimensional reduction: environmental auditory demand. The left panel displays the top 20 combinations of EMA responses to the “listening situation” item and their respective frequencies. The right panel shows the regrouping of EMA combinations into three categories which were given associated auditory demand scores: “Passive listening/ambient noise” (Least auditory demand, 0), “Active Listening” (Little auditory demand, 1), “Conversation 1 person” (Some auditory demand, 2) and “Conversation >1 person” (Most auditory demand, 3). Dashed lines connect EMA responses to their corresponding environmental auditory demand scores. Colors match each combination of EMA responses to the corresponding demand category.

Starting with the listening situation question, which had a total of seven answer choices, and given that each participant could choose multiple answers, a total of 61 combinations were generated from all submitted questionnaires. These 61 combinations were reassigned to four categories (“Conversation with >1 person,” “Conversation with 1 person,” “Active listening” and “Passive listening/Ambient noise”), which is in line with categorizations of environmental context discussed in the Common Sound Scenarios (CoSS) (Wolters et al., 2016). These four categories where furthermore ranked based on auditory demand from 3 (most auditory demand) to 0 (least auditory demand). Figure 2 provides an overview of this dimensional reduction.

**Figure 3.**
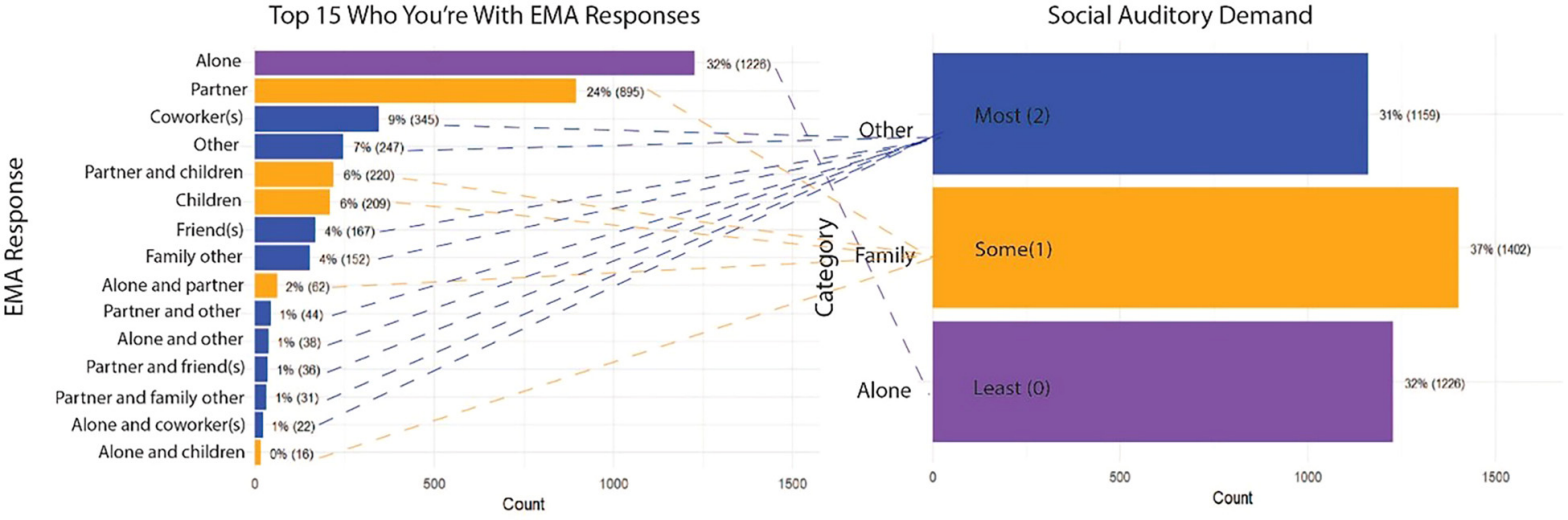
Dimensional reduction: social auditory demand. The left panel displays the top 15 combinations of EMA responses to the “who you’re with” item and their frequencies. The right panel shows the regrouping of EMA combinations into three categories which were given associated auditory demand scores: “Alone” (Least auditory demand, 0), “Family” (Medium auditory demand, 1), and “Other” (Most auditory demand, 2). Dashed lines connect EMA responses to their corresponding social auditory demand scores. Colors match each combination of EMA responses to the corresponding demand category.

A similar procedure was implemented for the “who you’re with” question to acquire the social auditory demand scores. Since participants could choose multiple choices from the seven answer options, this yielded many combinations of answer choices with different frequencies. In total, 28 combinations were yielded with “alone” being the most common selection (1226 instances). These 28 combinations were regrouped into three categories (“Other,” “Family,” and “Alone”) and where also given a rank order based on the auditory demand from 2 (Most auditory demand) to 0 (Least auditory demand). “Family” was given a medium level of auditory demand, given the familiarity and the likelihood of the frequency of encounter with a partner and/or children. “Other” was given the highest rank of auditory demand 2 (Most auditory demand) by assumption of less familiarity therefore making the auditory context higher burden (Souza et al., 2013). Figure 3 provides an overview of the dimensional reduction of this variable.

### Subjective Auditory Demand

**Figure 4.**
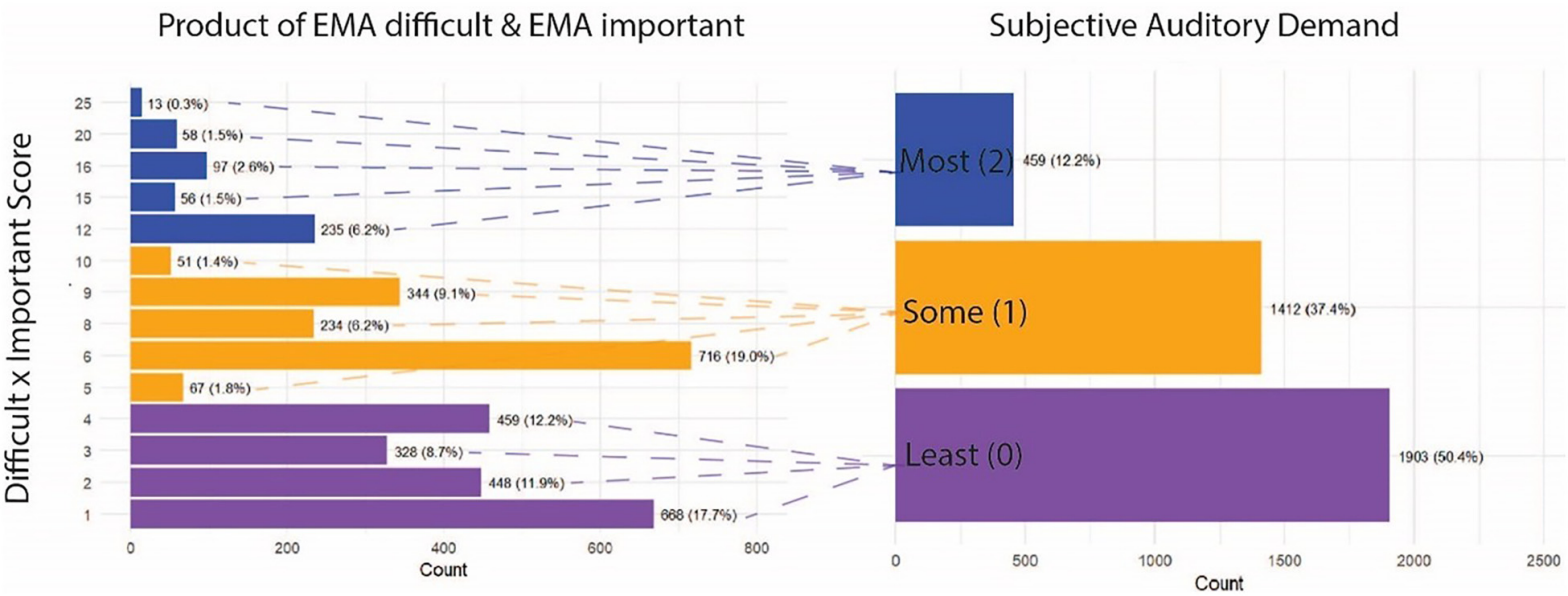
Dimensional reduction subjective auditory demand. The left panel shows the distribution of the product of two EMA questions (listening difficulty and importance to listen well). The right panel shows the regrouping of the products into subjective auditory demand scores of Least (0), Some (1), and Most (2). Dashed lines connect the Difficult × Important Score to their corresponding subjective auditory demand scores. Colors match each product to the corresponding subjective demand category.

The subjective auditory demand definition was based on the FUEL framework (Pichora-Fuller et al., 2016) that states that listening effort is a product of auditory task demand, or difficulty level and the motivation to perform well on the listening task. Difficulty level was estimated by the EMA item “How difficult was it to listen/hear well in the past 15 min?.” Motivation was estimated by the EMA item addressing importance to hear well (“How important was it to hear well in the past 15 min?”). Each of these questions was on a scale from 1 to 5. The product of these two EMA questions was taken and labeled as the “Difficult × Important score,” which ranged from a score of 1 to 25. The resulting scores were categorized into three levels ]least (0), some (1), most (2)] based on tertiles of the observed distribution. These corresponded to the subjective auditory demand rank from least (0) to most (2). The distribution of the product, or “Difficult × Important score,” as well as the associated final subjective auditory demand scores are shown in Figure 4.

### Between- and Within-Subject Correlation of the EMA Measures

To provide an overview of how the various EMA measures relate to each other, correlation matrices were generated using the R package “corrplot” (Wei & Simko, 2024). For between-subject correlations, we computed Pearson correlations across participants using their 5.5-day median values. For within-subject correlations, we first calculated correlation matrices for each individual participant and then took the median of these correlation coefficients across all participants to visualize the overall within-subject correlation structure. Then to compare the between and within subject matrices, we used Fisher *z*-tests based on variance-weighted pooling of individual within-subject Fisher *z* values (weights = *n*‒3). The resulting pooled within-subject correlations were back transformed for comparison with the between subject coefficients. An alpha of .007 was used to control for multiple testing (Li & Ji, 2005; Nyholt, 2004).

### Modeling PTA Effects on Average Auditory Demand

Before addressing the core aims of the study, linear regression models were run to assess the effect of hearing loss (PTA) on average auditory demand levels (environmental auditory demand, social auditory demand, and subjective auditory demand) using the “lme4” package in R.

### Modeling Auditory Demand Effects (and Moderation by PTA) on Listening Effort, Affect and Fatigue

To address the first aim of the study, we fit linear mixed effects models which were estimated using REML via the lme4 package in R whereby each time-varying auditory demand predictor (environmental, social and subjective) was decomposed into a within-person and between-person component. The within-person component was obtained by person-mean centering each participant's momentary scores, representing fluctuations around their own mean. The between-person component corresponded to each participant's mean across all measurements, grand-mean centered to facilitate interpretation. Both components were included in the models to distinguish within-person effects from between-person differences. These auditory demand models were used to separately predict listening effort, affect and fatigue. Furthermore, PTA (z-scored) was used as a Level-2 moderator of the within-person auditory demand effect [demand (within-person) × PTA (z-scored)] to assess whether the effect of auditory demand varies depending on degree of hearing loss (i.e., moderation by PTA). The models included the auditory demand × hearing loss (PTA) interaction as the primary test of our hypothesis, while other covariates (age, sex, sleep quality, sleep quantity at the between-subject level, and time of day at the within-subject level) were entered, in their original measurement units, as main effects only to control for confounding influences. Sex was entered as a binary predictor with female as the reference category. We considered results significant at *p < .*01 but provide effect sizes with confidence intervals throughout. Model assumptions were checked by visual inspection of residual and Q-Q plots. Figures were produced to visualize the interaction effects whereby regression lines were displayed at −1 SD, mean, and +1 SD of the PTA, corresponding to roughly 7 dB HL, 28 dB HL, and 48 dB HL in raw units (1 SD is ∼20 dB HL).

### Assessing the Mediating Role of Listening Effort and Affect on the (Moderated) Effect of Auditory Demand on Fatigue

To address the second aim of the study, we conducted multilevel mediation analyses to examine whether momentary listening effort and affect mediated the association between auditory demand and momentary fatigue and whether these indirect effects were moderated by hearing loss (PTA). Separate models were estimated for each demand definition (environmental, social, and subjective) and each mediator (effort and affect). The demand and mediator variables were person-mean centered to separate within-person and between-person variance. PTA was included as a moderator of the *a-path* (demand → mediator (either *listening effort* or *affect*)), allowing the strength of the indirect effect to vary across individuals with different hearing abilities.

Each mediation analysis was estimated using three linear mixed effects models using lme4. The first model (*Model A*) predicted the mediator (listening effort or affect) from momentary auditory demand, its person-mean component, PTA, and their interaction (demand*PTA), controlling for time of day, sleep quality, sleep hours, age and sex. The second model (*Model B*), predicted fatigue from both auditory demand definitions with the mediator using the same covariates, thereby estimating the *b-path* (mediator → fatigue) and the direct effect (*c’-path*) of auditory demand on fatigue after accounting for the mediator. The third model (*Model C*) predicted fatigue from auditory demand alone (plus covariates), representing the total effect (*c-path*) prior to including the mediator.

The indirect effect (a × b) was computed as a product of the estimated within-person *a* and *b* coefficients from Models A and B. Confidence intervals for the indirect effects were derived via Monte Carlo simulation (4,000 draws). Confidence intervals for fixed defects were based on Wald normal approximations.

## Results

### Study Population

Data from 130 participants was usable from the originally 136 recruited participants. Several participants (*N *= 6) were not able to execute the EMA procedure, had technical issues with their smartphone, or answered three prompts or less. The usable sample consisted of 91 females and 39 males, had a mean age of 58.3 years (SD 8.6). The average PTA was 27.4 dB HL (SD 20.1; Range: −5 to 93.75). A histogram of the hearing thresholds of the 130 participants is shown in Supplementary Figure 1. Out of the 130 participants, 68 reported wearing at least one hearing aid.

### EMA Overview

The table of the average EMA values for the EMA questions used for modeling, or for other analysis purposes is shown in Table 1. In Table 2, averages of the three demand definitions, derived from the EMA items are listed. Overall compliance rate was 75% and the averages across days were: Day 1: 83%, Day 2: 79%, Day 3: 75%, Day 4: 75%, Day 5: 70%, Day 6: 67%. Participants took an average of 67.7 s to fill in the survey.

**Table 1.** Summary Statistics From the EMA Measures Based on the Averages, per Participant.

EMA item	Mean	SD	Median	Min	Max
Fatigue	4.06	2.1	4.41	0	8.36
Listening effort	2.73	1.93	2.36	0	7.33
Listening importance	2.51	0.58	2.49	1.23	4.45
Listening difficulty	2.1	0.65	2	1	3.69
Feeling now (overall affect)	7.77	1.38	7.96	2.9	10
Positive affect	5.02	0.98	5.03	2.45	7
Negative affect	1.49	0.68	1.26	1	5.99

**Table 2.** Demand Definition Summary Statistics Derived From the EMA Measures and Based on the Averages, Per Participant.

Demand definition	Mean	SD	Median	Min	Max
Environmental auditory demand	1.5	0.42	1.49	0.49	2.68
Social auditory demand	0.98	0.35	0.95	0	1.86
Subjective auditory demand	0.63	0.39	0.55	0	1.8

**Figure 5.**
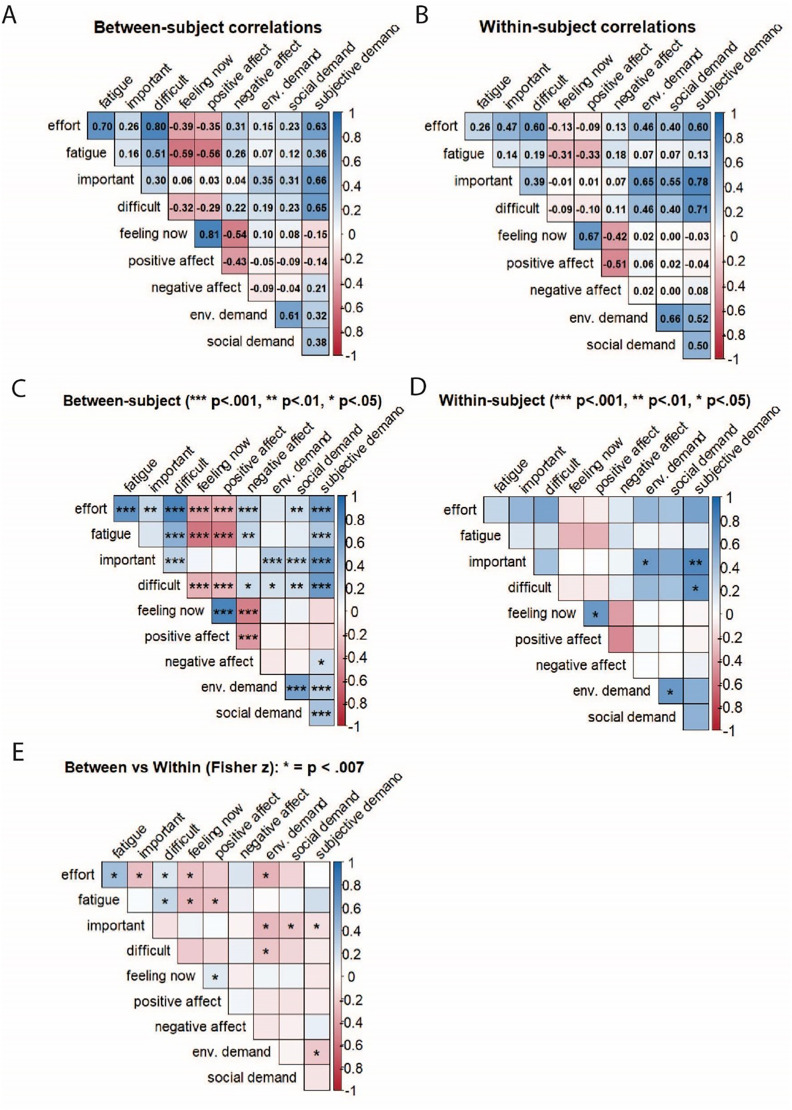
Between (panel A, C) and within-subject (panel B, D) correlograms of the EMA questions and the auditory demand groupings (environmental, social and subjective demand). Panels A and B display the full correlation matrices, while panels C and D highlight the significant correlations within those matrices with asterisks to reflect *p* < .001, *p* < .01, and *p* < .05. Panel E contrasts the between and within-subject correlation matrices using Fisher z-tests (alpha = .007). In Panel E, blue cells indicate stronger correlations at the between-subject level, red cells indicate stronger correlations at the within-subject level, and asterisks denote significant differences.

Between and within-subject correlograms are shown in Figure 5 and indicate that correlations between the various EMA measures, when comparing their median score between participants, were largely recaptured by the correlations across repeated assessments of the measures within a subject. Exceptions are noted in Panel E in the figure which indicate significant differences using Fisher z-tests. For example, the correlations between *fatigue* and *feeling now* are attenuated when looking at fluctuations within individuals compared to median levels across individuals. This may reflect a subject-specific response tendency, where negative statements on EMA items are more strongly endorsed by one person compared to another.

The correlograms (Figure 5) also show that both positive and negative affect were strongly correlated with the *feeling now* measure. To streamline analyses, we selected the *feeling now* measure as the primary indicator of overall affect. From this point forward we refer to the *feeling now* measure as *affect* in all the subsequent analyses and in the discussion.

Additional information about the spread of auditory demand instances per participants is available in Supplementary Table 8.

### Auditory Demand and Hearing Loss

Hearing loss (PTA) was significantly associated with average subjective auditory demand in daily life. The regression models revealed that more hearing loss (higher PTA) was associated with increased auditory subjective demand (β = 0.009, SE = 0.001, *t = *5.95, *p < .*001), accounting for 21.8% of the variance in subjective demand (*R*^2^ = 0.2178). However, hearing loss was not a strong predictor of environmental auditory demand (β = −0.0012, SE = 0.0018, *t = *−0.644, *p = .*521) or social auditory demand (β = −0.0021, SE = 0.0015, *t = *−1.356, *p = .*177).

### Auditory Demand Effects on Listening Effort, Affect and Fatigue

Linear mixed models showing the effect of each auditory demand definition on subjective listening effort, affect, and fatigue, as well as the interaction of the within-person auditory demand measure and PTA (to assess moderation) are listed in Table 3. Covariates such as sleep quality (0.01 < *p < .*001) and time of day (0.01 < *p < .*001) contributed significantly to the models, while age, sex and sleep duration had minimal effects. Listening effort (β =∼0.03), affect (β = ∼0.01), and fatigue (β = .13) increased with time of day, and those that reported higher sleep quality experienced lower levels of listening effort (β = ∼‒.38), more positive affect (β = ∼0.8), and lower levels of fatigue (β = ∼‒.85).

**Table 3.** Linear Regression Predicting Effort, Affect, and Fatigue, From Contextual (Environmental and Social) and Subjective Auditory Demand Definitions, as Well as Their Moderation by PTA. As a Reminder, Affect Is Derived From theFeeling Now Measure.

		Effort						Affect						Fatigue					
	Term	*β*	SE	Lower CI	Upper CI	*T*	*p*	*β*	SE	Lower CI	Upper CI	*t*	*p*	*β*	SE	Lower CI	Upper CI	*t*	*p*
Contextual auditory demand	Intercept	4.74	1.3	2.18	7.31	3.66	<.001	2.99	1.03	0.96	5.02	2.91	<.01	7.65	1.51	4.66	10.64	5.07	<.001
	Environmental demand (within-person)	0.77	0.02	0.72	0.82	31.47	<.001	0.03	0.02	−0.01	0.06	1.61	0.11	0.07	0.02	0.03	0.12	3.08	<.01
	PTA	1.19	0.13	0.93	1.46	8.9	<.001	−0.14	0.11	−0.35	0.07	−1.35	0.18	0.79	0.16	0.48	1.1	5.02	<.001
	Environmental demand (between-person)	0.97	0.31	0.37	1.58	3.17	<.01	0.1	0.24	−0.38	0.58	0.42	0.67	0.46	0.36	−0.25	1.16	1.28	0.20
	Time of day	0.02	0.01	0.01	0.03	3.34	<.001	0.01	0	0	0.02	2.96	<.01	0.13	0.01	0.12	0.14	20.98	<.001
	Sleep quality	−0.44	0.14	−0.71	−0.17	−3.27	<.01	0.77	0.11	0.56	0.98	7.21	<.001	−0.89	0.16	−1.2	−0.57	−5.63	<.001
	Sleep hours	0.14	0.12	−0.09	0.37	1.17	0.24	−0.06	0.09	−0.25	0.12	−0.7	0.49	0.13	0.14	−0.14	0.4	0.93	0.35
	Age	−0.02	0.02	−0.05	0.01	−1.44	0.15	0.03	0.01	0	0.05	2.21	<.05	−0.04	0.02	−0.07	0	−2.21	<.05
	Sex	−0.35	0.28	−0.9	0.2	−1.25	0.21	0	0.22	−0.44	0.43	−0.01	0.99	−0.8	0.32	−1.44	−0.15	−2.46	<.05
	Environmental demand (within-person) × PTA	0.34	0.02	0.29	0.39	14.19	<.001	−0.03	0.02	−0.07	0	−1.72	0.09	0.07	0.02	0.03	0.12	3.18	<.01
	Intercept	4.42	1.31	1.83	7	3.38	<.001	3.01	1.03	0.97	5.05	2.92	<.01	7.58	1.51	4.59	10.57	5.02	<.001
	Social demand (within-person)	0.96	0.04	0.89	1.03	25.94	<.001	0	0.03	−0.05	0.06	0.15	0.88	0.14	0.03	0.07	0.2	3.98	<.001
	PTA	1.22	0.14	0.95	1.48	9	<.001	−0.15	0.11	−0.36	0.06	−1.37	0.17	0.8	0.16	0.49	1.11	5.1	<.001
	Social demand (between-person)	1.11	0.38	0.36	1.86	2.94	<.01	−0.13	0.3	−0.71	0.46	−0.42	0.67	0.59	0.43	−0.27	1.45	1.36	0.18
	Time of day	0.04	0.01	0.02	0.05	5.65	<.001	0.01	0	0.01	0.02	3.03	<.01	0.13	0.01	0.12	0.14	21.31	<.001
	Sleep quality	−0.42	0.14	−0.69	−0.16	−3.13	<.01	0.79	0.11	0.58	1	7.4	<.001	−0.88	0.16	−1.19	−0.57	−5.64	<.001
	Sleep hours	0.13	0.12	−0.11	0.36	1.08	0.28	−0.07	0.09	−0.25	0.11	−0.74	0.46	0.12	0.14	−0.15	0.39	0.9	0.37
	Age	−0.02	0.02	−0.05	0.01	−1.38	0.17	0.03	0.01	0	0.05	2.09	<.05	−0.04	0.02	−0.07	0	−2.17	<.05
	Sex	−0.25	0.28	−0.81	0.31	−0.88	0.38	−0.02	0.22	−0.46	0.43	−0.07	0.95	−0.75	0.33	−1.39	−0.1	−2.28	<.05
	Social demand (within-person) × PTA	0.38	0.04	0.31	0.45	10.53	<.001	−0.02	0.03	−0.07	0.03	−0.74	0.46	0.09	0.03	0.03	0.16	2.78	<.01
Subjective auditory demand	Intercept	5.87	1.05	3.79	7.94	5.59	<.001	2.8	1.02	0.79	4.82	2.75	<.01	8.28	1.46	5.4	11.17	5.68	<.001
	Subjective demand (within-person)	1.81	0.04	1.73	1.89	44.64	<.001	−0.1	0.03	−0.17	−0.04	−3.05	<.01	0.38	0.04	0.3	0.46	8.83	<.001
	PTA	0.67	0.12	0.43	0.92	5.47	<.001	−0.05	0.12	−0.29	0.19	−0.43	0.67	0.52	0.17	0.18	0.85	3.01	<.01
	Subjective demand (between-person)	2.74	0.3	2.14	3.35	9	<.001	−0.47	0.3	−1.05	0.12	−1.58	0.12	1.42	0.42	0.58	2.25	3.34	<.01
	Time of day	0.03	0.01	0.02	0.04	4.68	<.001	0.02	0	0.01	0.02	3.24	<.01	0.13	0.01	0.12	0.14	21.13	<.001
	Sleep quality	−0.35	0.11	−0.57	−0.14	−3.31	<.01	0.78	0.1	0.57	0.98	7.49	<.001	−0.84	0.15	−1.14	−0.55	−5.67	<.001
	Sleep hours	−0.02	0.09	−0.2	0.17	−0.17	0.87	−0.05	0.09	−0.23	0.14	−0.5	0.62	0.05	0.13	−0.21	0.31	0.36	0.72
	Age	−0.03	0.01	−0.05	−0.01	−2.53	<.05	0.03	0.01	0	0.05	2.26	<.05	−0.04	0.02	−0.08	−0.01	−2.58	<.05
	Sex	−0.01	0.23	−0.46	0.44	−0.05	0.96	−0.07	0.22	−0.5	0.37	−0.3	0.77	−0.62	0.32	−1.25	0	−1.97	0.05
	Subjective demand (within-person) × PTA	0.37	0.04	0.3	0.45	9.54	<.001	−0.04	0.03	−0.11	0.02	−1.37	0.17	0.07	0.04	−0.01	0.15	1.78	0.08

### Moderation by PTA

Environmental demand (within-person) measure had a significant interaction effect with PTA on listening effort (β = 0.34, SE = 0.02, *t = *14.19, *p < .*001), indicating that individuals with higher PTA thresholds (i.e., more hearing loss) showed greater increases in listening effort when their momentary environmental demand was higher than usual. A similar finding was observed for the interaction term and its prediction of fatigue (β = 0.07, SE = 0.02, *t = *3.18, *p < .*01), supporting that individuals with higher (worse) PTA thresholds reported greater increases in fatigue as environmental demand increased more than usual. However, this interaction was not significant for affect (*p = .*09), suggesting that the within-person environmental demand effect on affect is not moderated by hearing loss level.

Similarly, the interaction between social auditory demand (within-person) and PTA significantly predicted listening effort (β = 0.38, SE = 0.04, *t = *10.53, *p < .*001), showing that higher PTA values were associated with a steeper increase in effort when social auditory demands increased. And once again, a similar finding was observed with the interaction term and its prediction of fatigue (β = 0.09, SE = 0.03, *t = *2.78, *p < .*01. For affect, however, social auditory demand's interaction with PTA was again not significant (*p = .*46), indicating that the influence of social auditory demand on affect was relatively stable across levels of hearing loss.

In contrast, subjective demand exhibited a more nuanced pattern. The interaction between within-person subjective demand and PTA significantly influenced listening effort (β = 0.37, SE = 0.04, *t = *9.54, *p < .*001) but not fatigue (β = 0.07, SE = 0.04, *t = *1.78, *p = .*08) or affect (β = −0.04, SE = 0.03, *t = *−1.37, *p = .*17). Specifically, individuals with higher PTA thresholds (worse hearing) reported increased listening effort as subjective demand increased.

**Figure 6.**
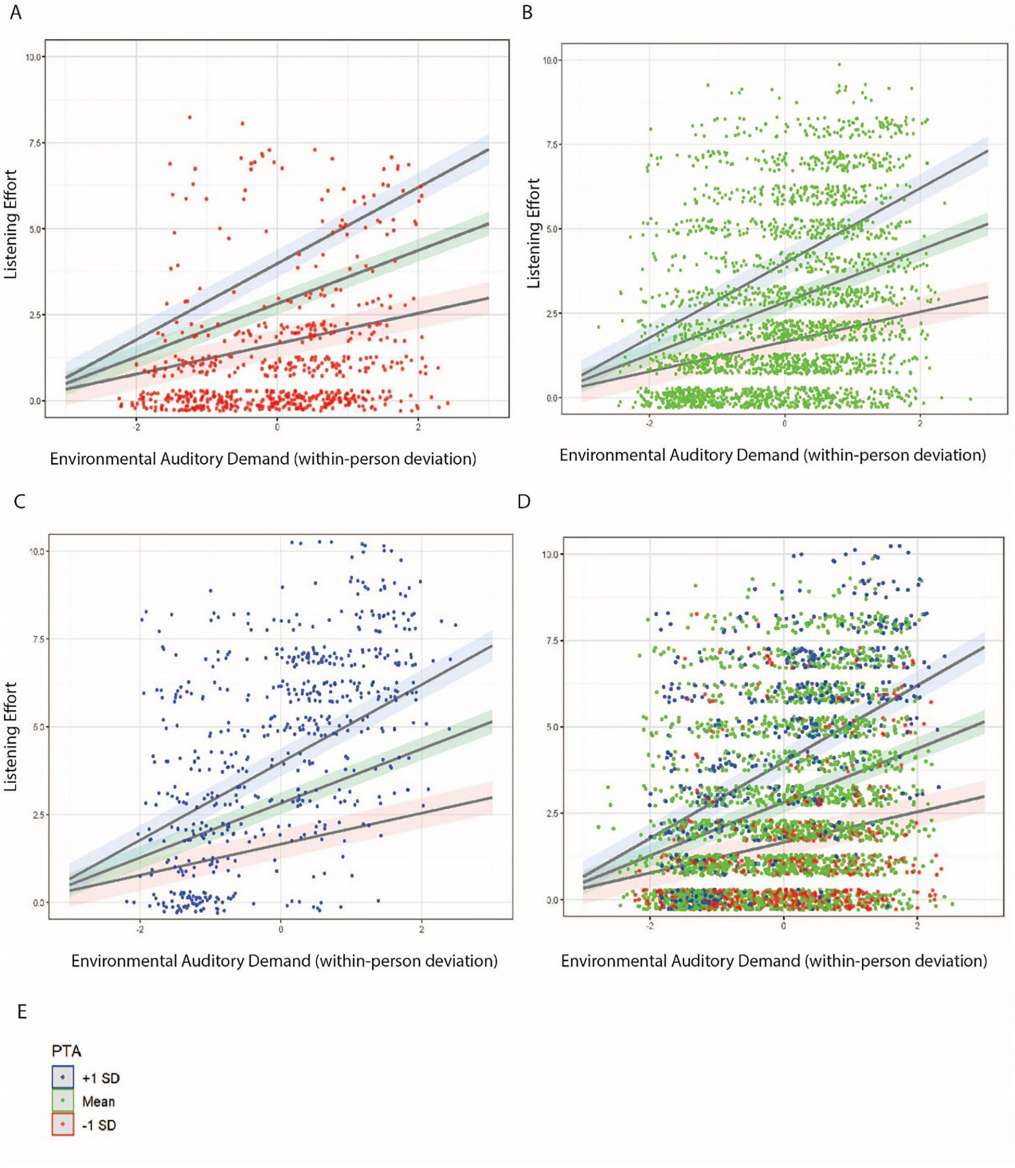
Linear regression plots illustrating the interaction of the environmental auditory demand definition and PTA to predict listening effort. Dots are jittered and reflect the raw data points. For illustration purposes, we have grouped the PTA values into three categories. Panels A, B and C show the same regression lines per PTA category, but each panel presents the individual dots from only one of these categories, for clarity. The red line represents the regression through the data points (red dots in panel A) from those with relatively low (good) PTAs. The green line represents the regression through the data points (green dots in panel B) from those with average PTAs. The blue line represents the regression through the data points (blue dots in panel C) from those with relatively high PTA thresholds (more severe hearing loss). Note that in the LME, PTA was included as a continuous measure. Panel D shows the combination of all points from Panels A through C, with the same fitted regression lines for each of the three PTA groupings. Panel E shows the legend for the various PTA groupings, used only for visualization.

**Figure 7.**
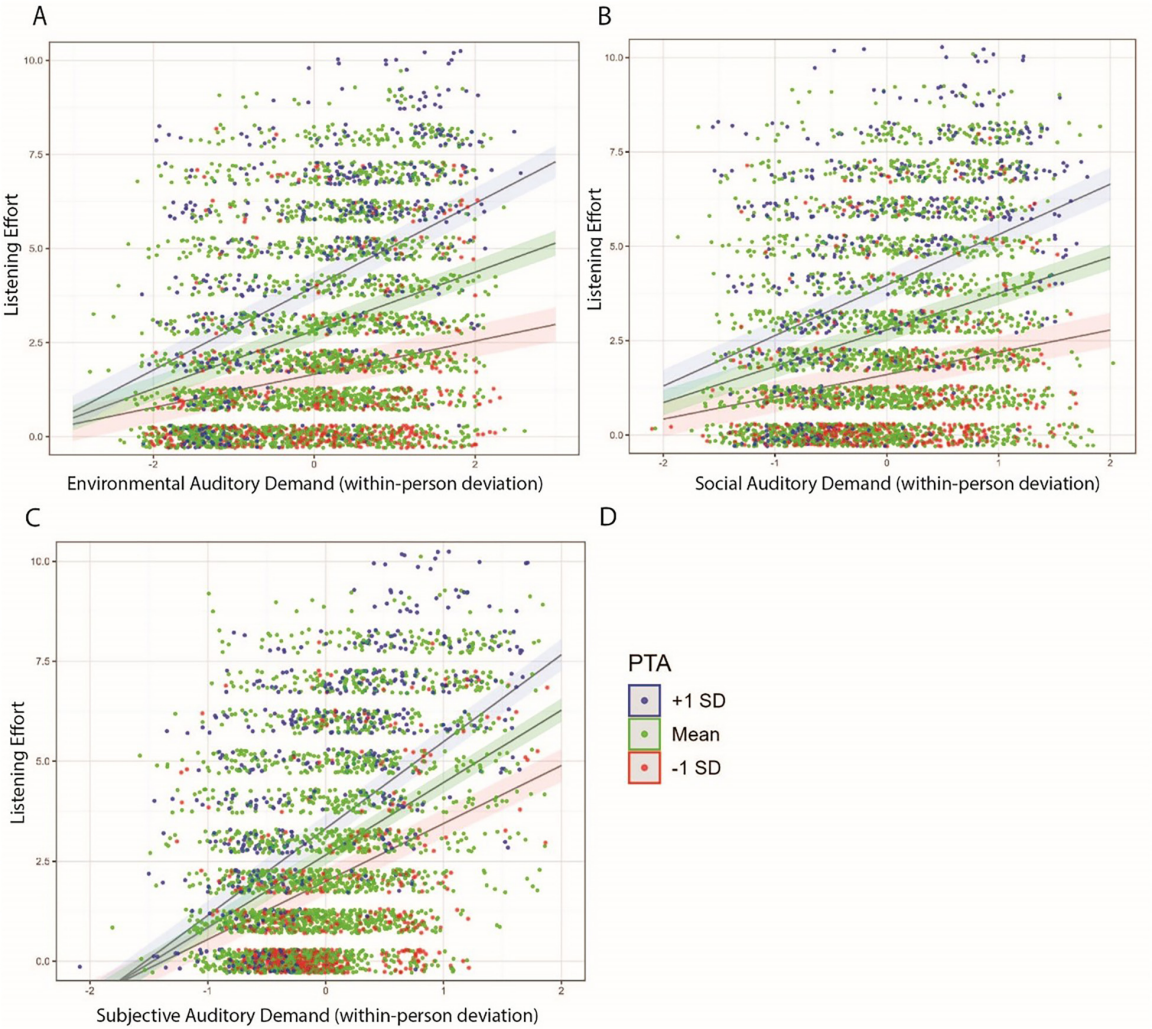
Linear regression plots illustrating the interaction of the various auditory demand definitions (Panel A: environmental; Panel B: social; Panel C: subjective) with PTA to predict listening effort. The PTA groupings are highlighted by the legend in Panel D. Dots are jittered and reflect the raw data points. Dot colors reflect PTA categories (which were created for illustration purposes only).

Figure 6 further illustrates the interaction of within-person environmental auditory demand and PTA on listening effort, with panels separating individuals into three PTA groups: those above +1 SD, those below −1 SD, and those within ±1 SD of the mean. Panel D combines these groups, revealing a steeper slope in listening effort for individuals with higher PTA thresholds as auditory demand increases. Figure 7 compares PTA interactions across all auditory demand definitions to predict listening effort, showing that while subjective demand exerted the strongest main effect on listening effort, the contextual auditory demand definitions (environmental and social), as well as subjective demand all demonstrated large interaction effects with PTA.

**Figure 8.**
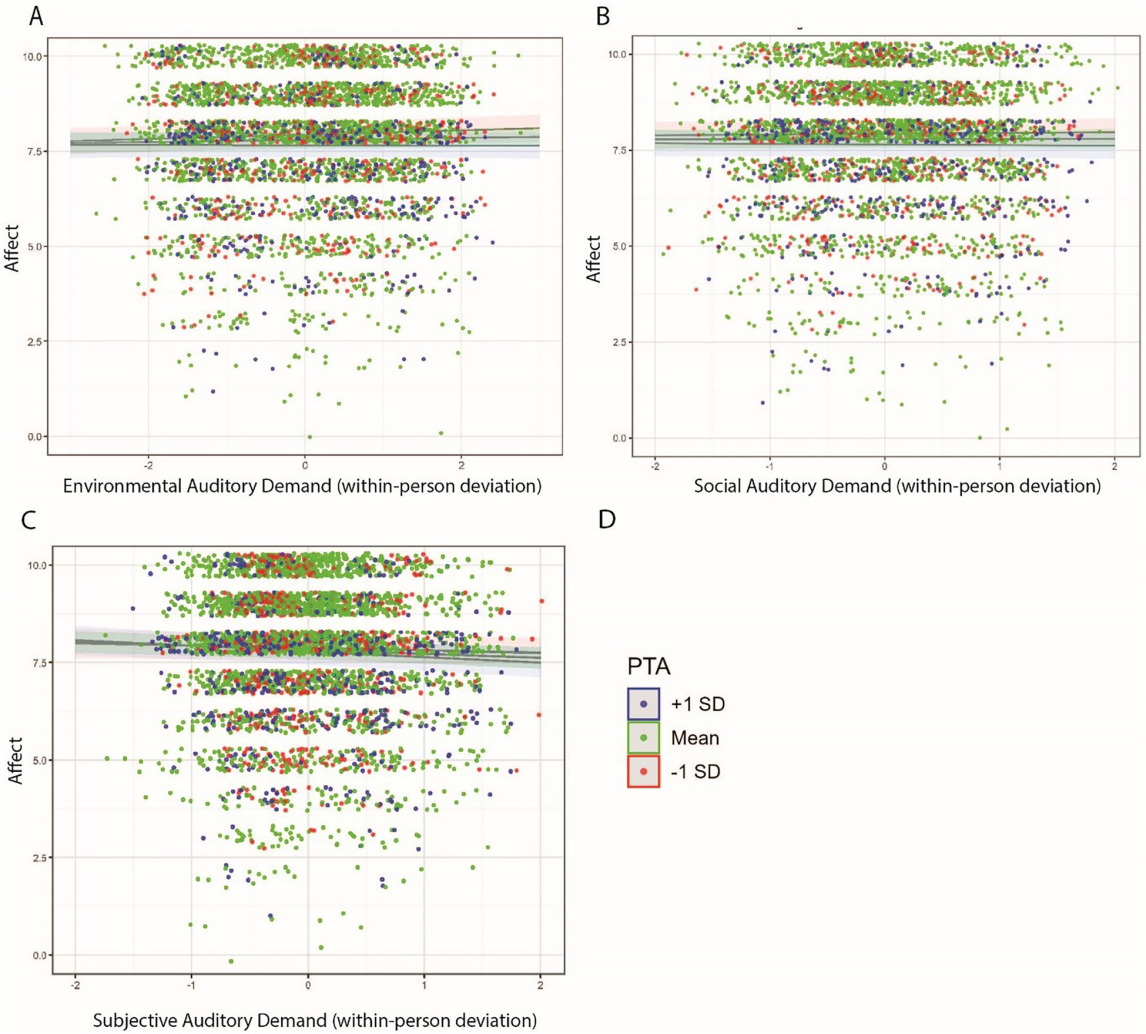
Linear regression plots highlighting the interaction of the various auditory demand definitions (Panel A: environmental; Panel B: social; Panel C: subjective) and PTA to predict affect. As a reminder, affect is derived from the *feeling now* measure. Dots are jittered and reflect the raw data points. Dot colors are grouped by PTA and highlighted in the legend in Panel D.

As previously noted, effects on affect were weaker overall and mainly limited to the subjective auditory demand definition. Figure 8 visualizes these findings for affect, underscoring the differential impact of demand types on affective experience among those with hearing loss.

**Figure 9.**
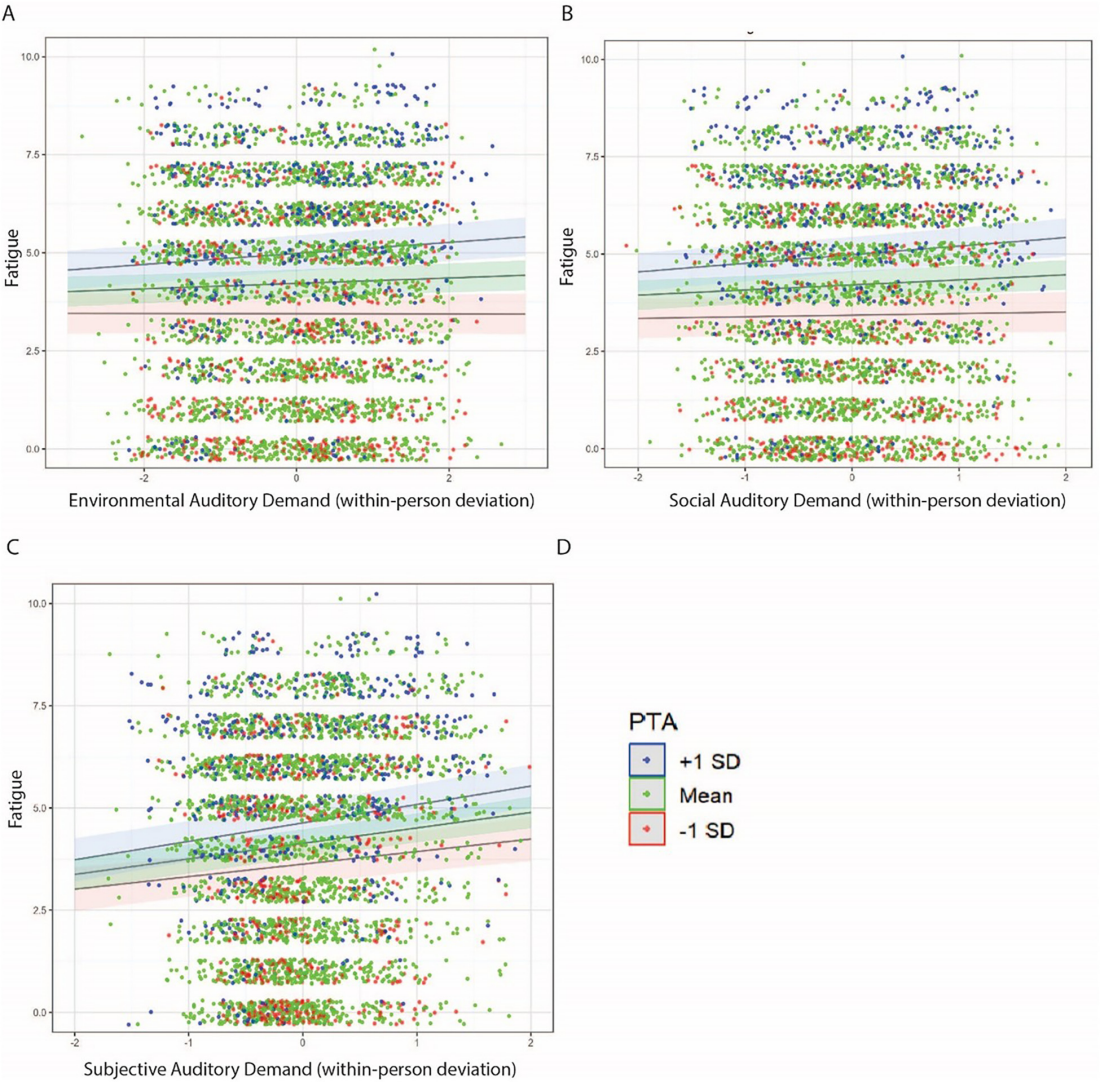
Linear regression plots highlighting the interaction of the various auditory demand definitions (Panel A: environmental; Panel B: social; Panel C: subjective) and PTA to predict fatigue. Dots are jittered and reflect the raw data points. Dot colors are grouped by PTA and highlighted in the legend in Panel D.

And lastly, Figure 9 visualizes the interaction effect of each auditory demand definition with PTA to predict fatigue. Here the contextual auditory demand definitions (environmental and social) demonstrated relatively larger interaction effects with PTA compared to subjective auditory demand

### Auditory Demand Effects on Fatigue With Mediation of Listening Effort

**Figure 10.**
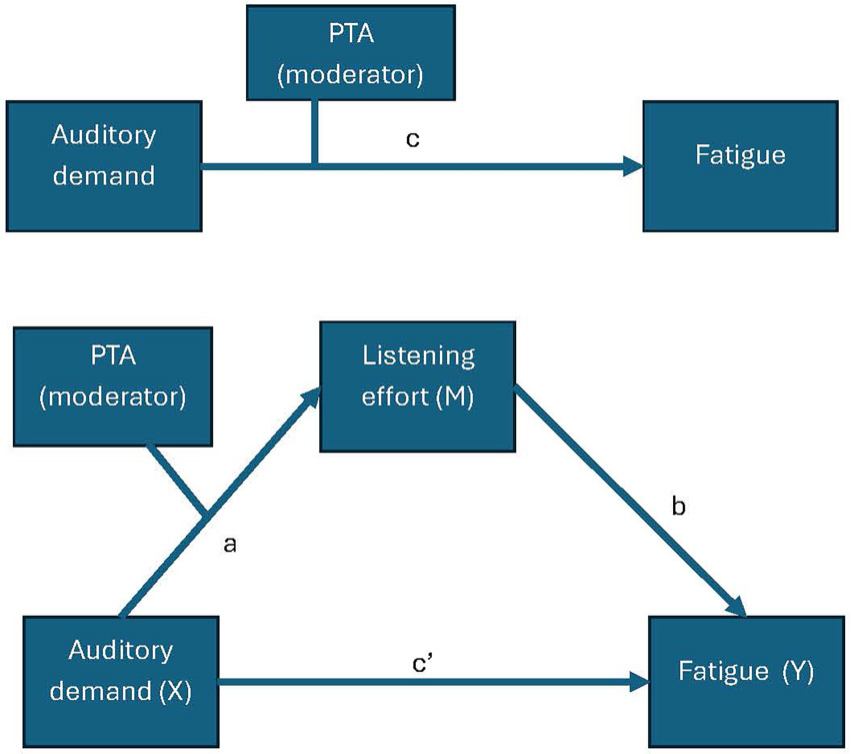
Schematic of the mediation analysis.

The schematic overview of the mediation analyses is shown in Figure 10 and the summary of the indirect, direct, total and moderated effects is listed in Table 4. For a full summary of the linear mixed effects model outputs, please see Supplementary Tables 2–7.

**Table 4.** Summary of Indirect, Direct, Total, and Moderated Effects of the Three Mediation Models (With the Three Demand Definitions)Using Listening Effort at the Mediator. This Panel Summarizes the Path-Specific Effects Derived from the Multilevel Mediation Models. thea-Path Represents the Effect of Auditory Demand on the Mediator (M; Listening Effort). The b-Path Represents the Effect of the Mediator onFatigue, Controlling for Auditory Demand. The Direct Effect (c′) Is the Effect of Auditory Demand on Fatigue After Accounting for ListeningEffort. The Total Effect (c) is the Effect of Auditory Demand on Fatigue Prior to Including the Mediator. the Indirect Effect (a × b) WasComputed as the Product of the Within-Person a and b Coefficients. The Final Row Shows the Moderation of the a-Path by Hearing Loss(PTA).

	Effect	Estimate	Lower CI	Upper CI	*p*
Environmental auditory demand	a (X→M)	0.77	0.72	0.82	<.001
	b (M→Y)	0.23	0.2	0.26	<.001
	Indirect a × b	0.18	0.15	0.2	
	Direct c′ (X→Y|M)	−0.1	−0.15	−0.05	<.001
	Total c (X→Y)	0.07	0.03	0.12	<.01
	a-path PTA moderation (environmental demand (within-person) × PTA)	0.34	0.29	0.39	<.001
Social auditory demand	a (X→M)	0.96	0.89	1.03	<.001
	b (M→Y)	0.21	0.18	0.24	<.001
	Indirect a × b	0.2	0.17	0.23	
	Direct c′ (X→Y|M)	−0.06	−0.13	0.01	0.08
	Total c (X→Y)	0.14	0.07	0.2	<.001
	a-path PTA moderation (environmental demand (within-person) × PTA)	0.38	0.31	0.45	<.001
Subjective auditory demand	a (X→M)	1.81	1.73	1.89	<.001
	b (M→Y)	0.2	0.16	0.23	<.001
	Indirect a × b	0.35	0.29	0.42	
	Direct c′ (X→Y|M)	0.02	−0.08	0.13	0.67
	Total c (X→Y)	0.38	0.3	0.46	<.001
	a-path PTA moderation (subjective demand (within-person) × PTA)	0.37	0.3	0.45	<.001

Moments of higher environmental auditory demand were associated with greater listening effort (*a* = 0.77, SE = 0.02, *p* < .001), and greater effort predicted higher fatigue (*b* = 0.23, SE = 0.02, *p* < .001) (Table 4). The indirect effect, estimated via Monte Carlo simulation (4,000 draws), was significant (*a* *×* *b* = 0.18, 95% CI = [0.15, 0.20]), indicating that effort explains a relatively large part of the demand–fatigue association. The total effect was positive (*c* = 0.07, 95% CI [0.03, 0.12], *p* < .01), whereas the direct effect became negative after accounting for effort (*c′* = −0.10, 95% CI [−0.15, −0.05], *p* < .001), a suppression pattern consistent with the association being largely carried by effort. Hearing loss (PTA) significantly moderated the *a*-path (β = 0.34, SE = 0.02, *p* < .001).

Moments of higher social auditory demand were associated with greater listening effort (*a* = 0.96, SE = 0.04, *p* < .001), and greater effort predicted higher fatigue (*b* = 0.21, SE = 0.01, *p* < .001). The indirect effect was significant (*a* *×* *b* = 0.20, 95% CI [0.17, 0.23]). The total effect was positive (*c* = 0.14, 95% CI [0.07, 0.20], *p* < .001), and the direct effect was attenuated and nonsignificant when effort was included (*c′* = −0.06, 95% CI [−0.13, 0.01], *p* = .08). PTA significantly moderated the *a*-path (β = 0.38, SE = 0.04, *p* < .001).

Furthermore, moments of higher subjective auditory demand were also associated with greater listening effort (*a* = 1.81, SE = 0.04, *p* < .001), and greater effort predicted higher fatigue (*b* = 0.20, SE = 0.02, *p* < .001). The indirect effect was significant and comparatively large (*a* *×* *b* = 0.35, 95% CI [0.29, 0.42]). The total effect was positive (*c* = 0.38, 95% CI [0.30, 0.46], *p* < .001), and the direct effect was reduced and nonsignificant controlling for effort (*c′* = 0.02, 95% CI [−0.08, 0.13], *p* = .67). PTA moderated the *a*-path (β = 0.37, SE = 0.04, *p* < .001).

Affect did not mediate the relationship between environmental or social auditory demand and fatigue, as demand was unrelated to affect in both models (see Supplementary Tables 5‒6). In contrast, subjective auditory demand predicted lower affect, which in turn predicted higher fatigue, yielding a significant indirect effect (*a* × *b* = 0.05, 95% CI [0.02, 0.07]) (see Supplementary Table 7). Across all models, affect remained a robust negative predictor of fatigue, and no significant moderation by hearing loss (PTA) was observed.

## Discussion

Previous research has demonstrated higher levels of fatigue among individuals with hearing loss, but few studies have systematically explored how momentary fatigue, listening effort, and affect are influenced by auditory demand in daily life. This study sought to fill this gap by employing an EMA protocol to repeatedly sample listening effort, fatigue and other contextual and subjective measures of auditory demand across diverse listening situations in adults with varying levels of hearing ability. Specifically, we examined the effect of auditory demand on listening effort, affect, and fatigue, with the expectation that these effects would be amplified by greater hearing loss. Additionally, we tested whether the relationship between auditory demand and fatigue, and its moderation by hearing loss, was mediated by listening effort and affect. Hearing ability was measured using PTA of the better ear, and constructs of contextual and subjective auditory demand were derived from EMA questions to investigate their distinct contributions to fatigue, listening effort, and affect.

### Auditory Demand Effects on Listening Effort, Affect, and Fatigue

Momentary increases in all three demand definitions (environmental, social, and subjective), were significant predictors of listening effort, as were higher levels of PTA. These demand effects primarily reflected within-person fluctuations such that when participant encountered more auditory demand than usual, they reported greater listening effort. Furthermore, the interaction of each auditory demand definition and PTA was also a significant predictor of listening effort. These findings support the notion that various components of auditory demand significantly influence the amount of listening effort reported and that this effect is amplified in individuals with greater hearing loss. This is in line with cognitive load theories and prior research indicating increased listening effort of those with hearing loss in challenging auditory situations (Hornsby, 2013; McGarrigle et al., 2014; Pichora-Fuller et al., 2016).

Affect was less influenced by contextual auditory demand and only subjective demand showed a weak association with more negative affect, but this was not moderated by PTA. This indicates that affect does not play a large role in the higher levels of fatigue induced by auditory demand reported by people with hearing loss. And although we observed generally high compliance in this study, it is worth noting that negative affect can reduce response likelihood and compliance has been shown to decline in the evening, when fatigue tends to peak (Rintala et al., 2020). This implies studies with lower compliance might underestimate the co-occurrence of high auditory demand and negative affect, as participants may be less likely to respond during such moments. However, given the relatively consistent compliance in this study, this potential bias is unlikely to have substantially affected the present findings. Despite the absence of significant interaction effects of demand and PTA in predicting affect, affect was still included in the mediation analysis, along with listening effort, as part of the hypothesized pathway linking auditory demand to fatigue.

Previous work has posited that auditory processing in challenging environments requires greater cognitive resources, leading to elevated fatigue (Alhanbali et al., 2017; Hornsby & Kipp, 2016; McGarrigle et al., 2014; Philips et al., 2023; Pichora-Fuller et al., 2016). Confirming these assumptions, the current study demonstrates that within-person fluctuations in daily-life contextual auditory demands, both environmental and social, were significant predictors of fatigue, with this effect amplified in participants with more hearing loss (higher PTA). In contrast, while subjective demand was a strong predictor of fatigue, its interaction with PTA did not reach significance, suggesting that self-perceived auditory demand increased fatigue similarly across participants, regardless of hearing level. Overall, these findings confirm that individuals with greater hearing loss experienced heightened fatigue due to the increased cognitive load associated with compensating for auditory challenges in real-world contexts.

This study's findings diverge somewhat from those of Burke and Naylor (2020), who, despite using EMA, found no consistent difference in momentary fatigue between individuals with and without hearing loss. Their results suggest that, within daily-life contexts, situational factors alone may not account for all differences in fatigue levels between hard of hearing and normal-hearing individuals. However, they did observe that participants who frequently encountered complex listening environments reported higher overall fatigue, indicating that cumulative exposure to demanding auditory situations can increase fatigue over time. In contrast, the current work here demonstrates a clear interaction between contextual auditory demand and hearing loss in predicting fatigue, with this effect being stronger among individuals with greater hearing loss. This difference could stem from variations in sample characteristics, sample size, measurement approaches, or the specific contextual variables examined. Unlike Burke and Naylor (2020), who focused on state, trait and momentary fatigue along with other listening context factors, the current study focused on different contextual variables of the listening scenery and used continuous levels of PTA as a moderator in the analyses, rather than a grouping structure (i.e., normal hearing vs. those with hearing loss, see Huizinga et al. 2025).

The present findings also extend the insights from lab-based studies, such as those by Hornsby (2013), who found that individuals with hearing loss experienced greater listening effort and fatigue under controlled, challenging listening conditions. By using tasks like speech-in-noise exercises and extended listening periods, Hornsby (2013) effectively simulated environments that demand high cognitive effort, providing valuable insight into how auditory challenges can increase fatigue (Hornsby, 2013). While these lab-based studies allow for precise control over listening conditions, real-world environments add layers of complexity that are difficult to replicate in the lab. By examining fatigue in daily life, the current work complements lab-based work by capturing how hearing loss and auditory demands interact dynamically across diverse and fluctuating real-world settings, offering a broader perspective on the fatigue experienced by individuals with hearing loss. And despite the intensive sampling schedule, participant compliance was high. Missingness is a common feature of EMA studies, yet our compliance rates were consistent with or higher than those typically reported, underscoring the reliability of the present data.

### Auditory Demand Effects on Fatigue With Mediating Role of Listening Effort and Affect

A critical finding of this study was that listening effort mediated the relationship between auditory demand and fatigue, whereas affect did not show a comparable mediating role. Across all three definitions of auditory demand, moments of higher demand were associated with increased listening effort, which in turn predicted greater fatigue. However, the nature of this mediation varied depending on the demand definition (environmental, social and subjective). For environmental auditory demand, the indirect effect through effort was positive and significant, whereas the direct effect reversed direction and remained significant, indicating inconsistent mediation and a suppression effect. This suggests that environmental demand primarily increased fatigue via effort, but when effort was controlled for, higher environmental demand was associated with slightly lower fatigue, potentially reflecting increase alertness or stimulation. For social auditory demand, a similar pattern emerged: the indirect effect was significant, but the direct effect reversed in sign and became nonsignificant, indicating that fatigue in this social framing was almost entirely explained by effort. This pattern supports the notion that fatigue in demanding listening context arises primarily from the cognitive effort invested in coping with those challenges.

From the perspective of cognitive load theory (Sweller et al., 2011), this indicates that the subjective experience of mental effort mediates the impact of task difficulty on fatigue. Another potential interpretation of the reversed and significant effect is to consider that, in less demanding environments, individuals might not exert as much effort, resulting in more passive listening or reduced attentiveness. This passive state could actually contribute to a subjective experience of fatigue, as reduced mental stimulation can sometimes lead to feelings of tiredness or lethargy, even if the demand of listening is lower (Hockey, 2013).

Finally, subjective auditory demand showed a partial mediation pattern, where both the total and indirect effects were positive and significant, while the direct effect became nonsignificant after including effort. This suggests that the subjective demand contributed to fatigue largely, but not exclusively, through listening effort.

These results refine the prior models by showing that contextual (environmental and social) and subjective auditory demand affect fatigue through partly distinct processes. This link between listening effort and fatigue is echoed in the remarks of McGarrigle et al. (2014), who reported that listening effort is closely linked to fatigue, particularly in hard of hearing individuals. Our results build on these studies by suggesting that in moments of high auditory demand in daily life, it is not merely the demands themselves, but the effortful engagement required to process these demands that contributes to fatigue.

In contrast, affect did not mediate the relationship between environmental or social auditory demand and fatigue, as demand was unrelated to affect in these models. Only subjective auditory demand predicted lower affect, which in turn predicted higher fatigue, yielding a small but significant indirect effect. Across all models, affect remained a strong negative predictor of fatigue, indicating that momentary mood plays a consistent but secondary role compared to listening effort, and that these processes were not moderated by hearing loss.

### Time of Day and Sleep Quality

Two covariates showed consistent significant effects across nearly all models: time of day and sleep quality. Throughout the course of the day, positive affect increased whereas listening effort also increased. Time of day was also a positive predictor of fatigue, a known and well-documented phenomenon (Kayser et al., 2022; Sletten et al., 2023). Because fatigue and listening effort increase with time of day, but negative affect decreases, this again suggests that listening effort rather than affect is a more likely correlate of fatigue.

At the same time there was a strong effect of sleep quality on fatigue, listening effort, and affect. Better sleep quality predicted lowered fatigue, lowered listening effort, and more positive affect. Accordingly, future research should focus on the protective effects of sleep quality for hearing loss-related complaints (Huizinga et al., 2025). Here, we used subjective reporting of sleep quality but adding polysomnography with EEG or even just actigraphy could help to better understand the sleep architecture in those with varied types and extents of hearing loss.

### Implications and Future Directions

Together, this study's findings suggest that reducing listening effort, rather than merely lowering auditory demand, could be key to mitigating fatigue in individuals with hearing problems. This perspective is supported by McGarrigle et al. (2014), who emphasized the importance of strategies aimed at alleviating cognitive load rather than just environmental adjustments. This is particularly relevant, given that social interaction, and communication are important staples of quality of life and simply removing the auditory demand completely or lowering it, could further alienate those with hearing loss. Future interventions might focus on optimizing hearing aids or providing training in communication strategies to reduce listening effort (Bess & Hornsby, 2014). This might also include the wider adoption of captioning and subtitling or supportive technology like microphones and streaming technology, for example, in classrooms, lecture halls, meeting rooms, and theatres (see European Federation of Hard of Hearing People (EFHOH) guidelines for resources). Additionally, exploring interventions that enhance cognitive resilience, such as mindfulness or cognitive training, could offer novel approaches to managing listening effort and fatigue (Bess & Hornsby, 2014). Furthermore, as research continues to assess listening effort and fatigue in more naturalistic settings, it would be worthwhile to incorporate findings from Davis et al. (2021), into an EMA procedure. For instance, Davis et al. (2021) point to the importance of collecting information about the number of speaker(s), familiarity of speaker(s), and vocal characteristics (such as speed of the talker). Including these contextual details could help to further disentangle sources of difficulty across specific situations, such as one-on-one conversations versus passive listening.

### Limitations

Notably, the questions regarding fatigue in this study focus on general fatigue, making it impossible to separate listening-related fatigue and more general momentary fatigue. Additionally, we did not screen for fatigue-related conditions in our participants, nor did we control for tinnitus or retirement status, which could have biased the results. Additionally, we did not control for hearing aid use, and while hearing aids are clinically relevant, their complex and individualized effects are better addressed in future studies specifically designed to examine them. We also did not control for personality traits, such as extraversion or neuroticism which could affect effort reporting and regulation (Eysenck, 1990; Eysenck & Derakshan, 2011).

We intentionally retained participants who primarily experienced low-demand contexts, as our aim was to capture the full range of real-life listening situations and not bias the sample toward participants with more socially or environmentally demanding lifestyles, though this may have reduced within-person variability for some individuals. Additionally, the subjective auditory demand was regrouped into three levels, from 0 to 2, based on tertiles of the distribution, as no established cutoffs exist for this combined difficulty × importance score. This categorization enhances interpretability but may limit generalizability to other samples with different score distributions.

A further consideration concerns the misalignment in temporal framing between measures of listening effort, affect, and fatigue. Following common EMA conventions, affect was assessed “in the moment” to minimize recall bias and ensure ecological validity (van der Mee et al., 2022). In contrast, listening effort was assessed over the preceding 15 min. This difference in temporal focus complicates direct comparisons between affect and effort responses, although it reflects established methodological practices in each domain.

## Conclusion

Our findings demonstrated that both contextual and subjective auditory demand definitions predict listening effort, with higher auditory demand leading to significantly greater effort at the within-person level. Importantly, this relationship was amplified in individuals with hearing loss, reflecting the heightened cognitive load required to process auditory information under adverse conditions. Mediation analyses revealed that listening effort plays a notable role in the relationship between auditory demand and fatigue, although the underlying patterns differed across the demand definitions. Affect, by contrast, was only related to subjective auditory demand, was not moderated by hearing loss and did not play a comparable mediating role in the demand—fatigue relationship. These findings underscore the central role of cognitive processes, specifically listening effort, in driving the fatigue experienced by individuals with hearing loss during periods of high auditory demand.

Furthermore, this study confirms hypotheses derived from prior research using static surveys and clinical interviews, reinforcing the notion that hearing loss contributes to fatigue through increased listening effort rather than emotional distress. By employing EMA, we extend these insights to dynamic, real-world settings, offering a more comprehensive understanding of how auditory and environmental factors contribute to fatigue in daily life. Future work should focus on interventions targeting listening effort to mitigate fatigue, such as optimizing hearing aids or developing cognitive strategies to reduce the burden of auditory processing.

## Supplemental Material

sj-docx-1-tia-10.1177_23312165251413329 - Supplemental material for The Effects of Daily Life Auditory Demands on Listening Effort, Affect, and Fatigue as a Function of Hearing Loss Supplemental material, sj-docx-1-tia-10.1177_23312165251413329 for The Effects of Daily Life Auditory Demands on Listening Effort, Affect, and Fatigue as a Function of Hearing Loss by Nicole A. Huizinga, Laura Keur-Huizinga, Adriana A. Zekveld, Sophia E. Kramer and Eco J.C. de Geus in Trends in Hearing
