# Supplementary material for: The Effects of Daily Life Auditory Demands on Listening Effort, Affect, and Fatigue as a Function of Hearing Loss
Source: Trends Hear. 2026 Mar 3;30:23312165251413329. doi: 10.1177/23312165251413329 (PMC12966542; doi:10.1177/23312165251413329)
Supplement: sj-docx-1-tia-10.1177_23312165251413329 - Supplemental material for The Effects of Daily Life Auditory Demands on Listening Effort, Affect, and Fatigue as a Function of Hearing Loss [file sj-docx-1-tia-10.1177_23312165251413329.docx]

**Table 1. Full set of EMA questions**

| Question (Dutch) | Question (English) | Time point of reference for item | Scale of answer | Scale of answer detail (Dutch) | Scale of answer detail (English) |
| --- | --- | --- | --- | --- | --- |
| 1. Hoe voelt u zich nu? Geef aan hoe u zich op dit moment voelt. | 1. How are you feeling now? Please indicate how you are feeling at the moment. | At this moment | 11-point likert scale | 0 tot 10 ('Erg slecht' tot 'Erg goed') | 0 tot 10 ('Very bad' to 'Very good') |
| 2. Ik voel me ontspannen... | 2. I feel relaxed... | At this moment | 7-point likert scale | 1-7 ('Niet' tot 'Zeer') | 1-7 ('Not' to 'A lot/Much') |
| 3. Ik voel me onzeker… | 3. I feel insecure... | At this moment | 7-point likert scale | 1-7 ('Niet' tot 'Zeer') | 1-7 ('Not' to 'A lot/Much') |
| 4. Ik voel me opgewekt… | 4. I feel excited… | At this moment | 7-point likert scale | 1-7 ('Niet' tot 'Zeer') | 1-7 ('Not' to 'A lot/Much') |
| 5. Ik voel me eenzaam… | 5. I feel lonely… | At this moment | 7-point likert scale | 1-7 ('Niet' tot 'Zeer') | 1-7 ('Not' to 'A lot/Much') |
| 6. Ik voel me angstig… | 6. I feel anxious… | At this moment | 7-point likert scale | 1-7 ('Niet' tot 'Zeer') | 1-7 ('Not' to 'A lot/Much') |
| 7. Ik voel me enthousiast… | 7. I feel enthusiastic… | At this moment | 7-point likert scale | 1-7 ('Niet' tot 'Zeer') | 1-7 ('Not' to 'A lot/Much') |
| 8. Ik voel me geïrriteerd… | 8. I feel irritated… | At this moment | 7-point likert scale | 1-7 ('Niet' tot 'Zeer') | 1-7 ('Not' to 'A lot/Much') |
| 9. Ik voel me tevreden… | 9. I feel satisfied… | At this moment | 7-point likert scale | 1-7 ('Niet' tot 'Zeer') | 1-7 ('Not' to 'A lot/Much') |
| 10. Ik voel me somber… | 10. I feel sad… | At this moment | 7-point likert scale | 1-7 ('Niet' tot 'Zeer') | 1-7 ('Not' to 'A lot/Much') |
| 11. Met wie was je? | 11. Who were you with? | Last 15 minutes | categorical | 1-7 ((1) Alleen; (2) Partner; (3) Kinderen; (4) Een of meerdere collega's; (5) Familie (behalve uw partner en kinderen); (6) Vriend(en); (7) Anders) | 1-7 ((1) Alone; (2) Partner; (3) Children; (4) One or more colleagues; (5) Family (besides your partner and children); (6) Friend(s); (7) Other) |
| 12. Wat was de luistersituatie? | 12. What was the listening situation? | Last 15 minutes | categorical | 1-7 ((1) Gesprek met 1 persoon; (2) Gesprek met meer dan 1 persoon; (3) Telefoongesprek; (4) Actief luisteren naar een spreker (live); (5) Actief luisteren naar TV, radio, etc.; (6) Omgevingsgeluid; (7) Passief luisteren, geen actieve luistertaak) | 1-7 ((1) Conversation with 1 person; (2) Conversation with more than 1 person; (3) Phone conversation; (4) Actively listening to a speaker (live); (5) Actively listening to TV, radio, etc.; (6) Ambient noise; (7) Passive listening, not an active listening task) |
| 13. How moeilijk was het om te horen/luisteren in de afgelopen 15 minuten? | 13. How difficult was it to hear/listen in the past 15 minutes? | Last 15 minutes | categorical | 1-5 ((1) zeer makkelijk; (2) makkelijk; (3) niet makkelijk en niet moeilijk; (4) moeilijk; (5) zeer moeilijk) | 1-5 ((1) very easy; (2) easy; (3) not easy and not difficult; (4) difficult; (5) very difficult) |
| 14. Hoe belangrijk was het voor u dat u goed kon horen in de luistersituaties in de afgelopen 15 minuten? | 14. How important was it to hear well in the past 15 minutes? | Last 15 minutes | categorical | 1-5 ((1) niet belangrijk; (2) een beetje belangrijk; (3) belangrijk; (4) erg belangrijk; (5) zeer belangrijk) | 1-5 ((1) not important; (2) somewhat important; (3) important; (4) very important; (5) extremely important) |
| 15. Hoeveel inspanning kostte het om te luisteren in de luistersituaties in de afgelopen 15 minuten? | 15. How much effort did it take to listen in the lsitening situations in the past 15 minutes? | Last 15 minutes | 11-point likert scale | 0-10 ('geen inspanning' tot 'extreem hoge inspanning') | 0-10 ('no effort' to 'extremely high effort') |
| 16. Heb u de agfelopen 15 minuten een koptelefoon gebruikt? | 16. Have you used headphones in the past 15 minutes? | Last 15 minutes | binary | Ja/Nee | Yes/No |
| 17. Heef u de afgelopen 15 minuten hoortoestellen gebruikt? | 17. Have you used a hearing aid in the past 15 minutes? | Last 15 minutes | binary | Ja/Nee | Yes/No |
| 18. Beoordeel uw vermoeidheid door het cifter te slecteren dat op dit moment uw vermoeidheid het beste beschrijft. | 18. Rate your fatigue by selecting the number that best describes your fatigue at the moment. | At this moment | 11-point likert scale | 0-10 ('geen vermoeidheid' tot 'extreme vermoeidheid') | 0-10 ('no fatigue' to 'extreme fatigue') |

**Figure 1.** Distribution of Hearing Thresholds based on the PTA of the better ear.


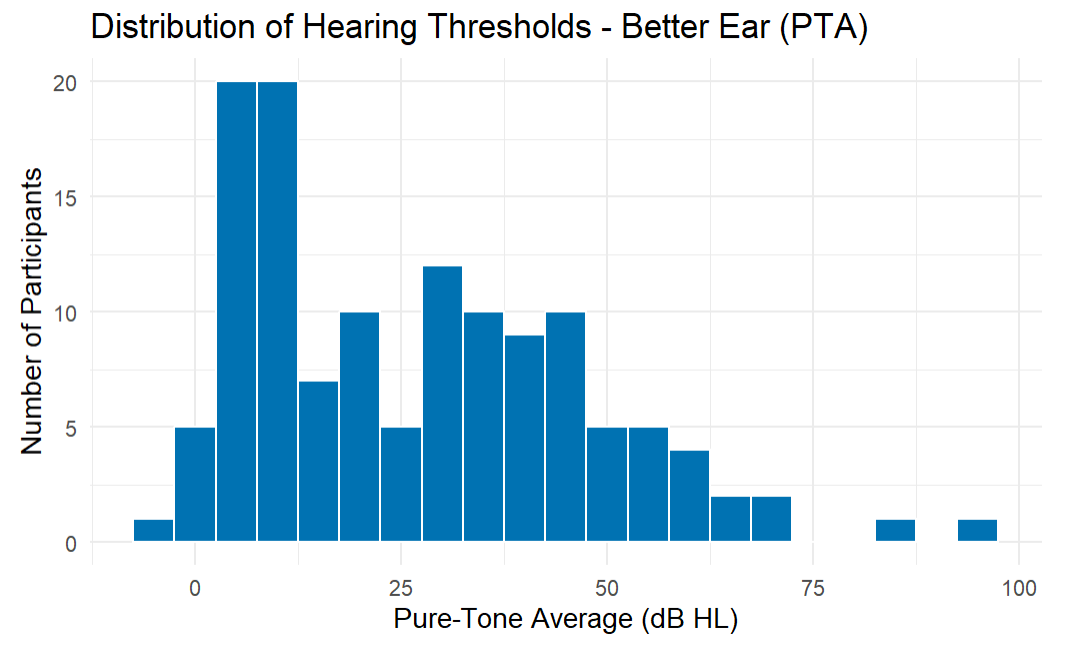


**Supplementary Table 2** *Multilevel mediation model testing the indirect effect of environmental auditory demand on fatigue through listening effort, moderated by hearing loss (PTA).*

**Table 2A.** Fixed effects from Models A–C. Model A predicts listening effort from auditory demand (a-path). Model B predicts fatigue from both auditory demand and listening effort (b- and c′-paths). Model C predicts fatigue from auditory demand alone (c-path).

|  |  | **Model A: Demand 🡪 Listening Effort (*a-path*)** | | | | | | **Model B: Fatigue with Effort (*b-*and *c’-paths*)** | | | | | | **Model C: Demand 🡪 Fatigue (*c-path*)** | | | | | |
| --- | --- | --- | --- | --- | --- | --- | --- | --- | --- | --- | --- | --- | --- | --- | --- | --- | --- | --- | --- |
|  | **Term** | ***β*** | **SE** | **Lower CI** | **Upper CI** | **t** | **p** | ***β*** | **SE** | **Lower CI** | **Upper CI** | **t** | **p** | ***β*** | **SE** | **Lower CI** | **Upper CI** | **t** | **p** |
| **Environmental Auditory Demand** | Intercept | 4.74 | 1.3 | 2.18 | 7.31 | 3.66 | <.001 | 3.91 | 1.23 | 1.48 | 6.34 | 3.19 | <.01 | 7.65 | 1.51 | 4.66 | 10.64 | 5.07 | <.001 |
|  | Environmental demand (within-person) | 0.77 | 0.02 | 0.72 | 0.82 | 31.47 | <.001 | -0.1 | 0.03 | -0.15 | -0.05 | -3.99 | <.001 | 0.07 | 0.02 | 0.03 | 0.12 | 3.08 | <.01 |
|  | PTA | 1.19 | 0.13 | 0.93 | 1.46 | 8.9 | <.001 | -0.11 | 0.15 | -0.42 | 0.19 | -0.74 | 0.46 | 0.79 | 0.16 | 0.48 | 1.1 | 5.02 | <.001 |
|  | Environmental demand (between-person) | 0.97 | 0.31 | 0.37 | 1.58 | 3.17 | <.01 | -0.27 | 0.28 | -0.84 | 0.29 | -0.96 | 0.34 | 0.46 | 0.36 | -0.25 | 1.16 | 1.28 | 0.20 |
|  | Hour | 0.02 | 0.01 | 0.01 | 0.03 | 3.34 | <.001 | 0.13 | 0.01 | 0.11 | 0.14 | 20.79 | <.001 | 0.13 | 0.01 | 0.12 | 0.14 | 20.98 | <.001 |
|  | Sleep quality | -0.44 | 0.14 | -0.71 | -0.17 | -3.27 | <.01 | -0.55 | 0.13 | -0.8 | -0.31 | -4.41 | <.001 | -0.89 | 0.16 | -1.2 | -0.57 | -5.63 | <.001 |
|  | Sleep hours | 0.14 | 0.12 | -0.09 | 0.37 | 1.17 | 0.24 | 0.02 | 0.1 | -0.18 | 0.23 | 0.22 | 0.82 | 0.13 | 0.14 | -0.14 | 0.4 | 0.93 | 0.35 |
|  | Age | -0.02 | 0.02 | -0.05 | 0.01 | -1.44 | 0.15 | -0.02 | 0.01 | -0.05 | 0 | -1.67 | 0.10 | -0.04 | 0.02 | -0.07 | 0 | -2.21 | <.05 |
|  | Sex | -0.35 | 0.28 | -0.9 | 0.2 | -1.25 | 0.21 | -0.54 | 0.25 | -1.03 | -0.04 | -2.16 | <.05 | -0.8 | 0.32 | -1.44 | -0.15 | -2.46 | <.05 |
|  | Environmental demand (within-person) × PTA | 0.34 | 0.02 | 0.29 | 0.39 | 14.19 | <.001 | 0 | 0.02 | -0.05 | 0.04 | -0.2 | 0.84 | 0.07 | 0.02 | 0.03 | 0.12 | 3.18 | <.01 |
|  | Listening effort (within-person) |  |  |  |  |  |  | 0.23 | 0.02 | 0.2 | 0.26 | 14.63 | <.001 |  |  |  |  |  |  |
|  | Listening effort (between-person) |  |  |  |  |  |  | 0.75 | 0.08 | 0.59 | 0.91 | 9.31 | <.001 |  |  |  |  |  |  |

**Table 2B*.*** Summary of indirect, direct, total, and moderated effects. This panel summarizes the path-specific effects derived from Models A–C in Panel A. The a-path represents the effect of auditory demand on the mediator (listening effort). The b-path represents the effect of the mediator on fatigue, controlling for auditory demand. The direct effect (c′) is the effect of auditory demand on fatigue after accounting for listening effort. The total effect (c) is the effect of auditory demand on fatigue prior to including the mediator. The indirect effect (a × b) was computed as the product of the within-person a and b coefficients. The final row shows the moderation of the a-path by hearing loss (PTA). Confidence intervals for fixed effects are Wald normal approximations; those for indirect effects are based on Monte Carlo simulations.

| **Effect** | **Estimate** | **2.5%** | **97.5%** | **p-value** |
| --- | --- | --- | --- | --- |
| a (X→M) | 0.77 | 0.72 | 0.82 | <.001 |
| b (M→Y) | 0.23 | 0.2 | 0.26 | <.001 |
| Indirect a×b | 0.18 | 0.15 | 0.2 |  |
| Direct c′ (X→Y\|M) | -0.1 | -0.15 | -0.05 | <.001 |
| Total c (X→Y) | 0.07 | 0.03 | 0.12 | <.01 |
| a-path PTA moderation (environmental demand (within-person) × PTA) | 0.34 | 0.29 | 0.39 | <.001 |

**Table 3.** *Multilevel mediation model testing the indirect effect of social auditory demand on fatigue through listening effort, moderated by hearing loss (PTA).*

**Table 3A.** Fixed effects from Models A–C. Model A predicts listening effort from auditory demand (a-path). Model B predicts fatigue from both auditory demand and listening effort (b- and c′-paths). Model C predicts fatigue from auditory demand alone (c-path).

|  |  | **Model A (Demand -> Effort)** | | | | | | **Model B Fatigue with Effort** | | | | | | **Model C Fatigue total** | | | | | |
| --- | --- | --- | --- | --- | --- | --- | --- | --- | --- | --- | --- | --- | --- | --- | --- | --- | --- | --- | --- |
|  | **Term** | ***β*** | **SE** | **Lower CI** | **Upper CI** | **t** | **p** | ***β*** | **SE** | **Lower CI** | **Upper CI** | **t** | **p** | ***β*** | **SE** | **Lower CI** | **Upper CI** | **t** | **p** |
| **Contextual Auditory Demand** | Intercept | 4.42 | 1.31 | 1.83 | 7 | 3.38 | <.001 | 3.98 | 1.23 | 1.55 | 6.42 | 3.24 | <.01 | 7.58 | 1.51 | 4.59 | 10.57 | 5.02 | <.001 |
|  | Social demand (within-person) | 0.96 | 0.04 | 0.89 | 1.03 | 25.94 | <.001 | -0.06 | 0.04 | -0.13 | 0.01 | -1.73 | 0.08 | 0.14 | 0.03 | 0.07 | 0.2 | 3.98 | <.001 |
|  | PTA | 1.22 | 0.14 | 0.95 | 1.48 | 9 | <.001 | -0.11 | 0.15 | -0.42 | 0.2 | -0.71 | 0.48 | 0.8 | 0.16 | 0.49 | 1.11 | 5.1 | <.001 |
|  | Social demand (between person) | 1.11 | 0.38 | 0.36 | 1.86 | 2.94 | <.01 | -0.24 | 0.35 | -0.93 | 0.45 | -0.69 | 0.49 | 0.59 | 0.43 | -0.27 | 1.45 | 1.36 | 0.18 |
|  | Hour | 0.04 | 0.01 | 0.02 | 0.05 | 5.65 | <.001 | 0.12 | 0.01 | 0.11 | 0.14 | 20.56 | <.001 | 0.13 | 0.01 | 0.12 | 0.14 | 21.31 | <.001 |
|  | Sleep quality | -0.42 | 0.14 | -0.69 | -0.16 | -3.13 | <.01 | -0.57 | 0.12 | -0.81 | -0.32 | -4.54 | <.001 | -0.88 | 0.16 | -1.19 | -0.57 | -5.64 | <.001 |
|  | Sleep hours | 0.13 | 0.12 | -0.11 | 0.36 | 1.08 | 0.28 | 0.03 | 0.1 | -0.18 | 0.23 | 0.26 | 0.80 | 0.12 | 0.14 | -0.15 | 0.39 | 0.9 | 0.37 |
|  | Age | -0.02 | 0.02 | -0.05 | 0.01 | -1.38 | 0.17 | -0.02 | 0.01 | -0.05 | 0 | -1.67 | 0.10 | -0.04 | 0.02 | -0.07 | 0 | -2.17 | <.05 |
|  | Sex | -0.25 | 0.28 | -0.81 | 0.31 | -0.88 | 0.38 | -0.56 | 0.25 | -1.06 | -0.06 | -2.23 | <.05 | -0.75 | 0.33 | -1.39 | -0.1 | -2.28 | <.05 |
|  | Social demand (within-person) × PTA | 0.38 | 0.04 | 0.31 | 0.45 | 10.53 | <.001 | 0.01 | 0.03 | -0.05 | 0.08 | 0.42 | 0.67 | 0.09 | 0.03 | 0.03 | 0.16 | 2.78 | <.01 |
|  | Listening effort (within-person) |  |  |  |  |  |  | 0.21 | 0.01 | 0.18 | 0.24 | 14.05 | <.001 |  |  |  |  |  |  |
|  | Listening effort (between person) |  |  |  |  |  |  | 0.75 | 0.08 | 0.59 | 0.91 | 9.24 | <.001 |  |  |  |  |  |  |

**Table 3B*.*** Summary of indirect, direct, total, and moderated effects. This panel summarizes the path-specific effects derived from Models A–C in Table 2, Panel A. The a-path represents the effect of social auditory demand on the mediator (listening effort). The b-path represents the effect of the mediator on fatigue, controlling for auditory demand. The direct effect (c′) is the effect of social auditory demand on fatigue after accounting for listening effort. The total effect (c) is the effect of social auditory demand on fatigue prior to including the mediator. The indirect effect (a × b) was computed as the product of the within-person a and b coefficients. The final row shows the moderation of the a-path by hearing loss (PTA). Confidence intervals for fixed effects are Wald normal approximations; those for indirect effects are based on Monte Carlo simulations.

| **Effect** | **Estimate** | **2.5%** | **97.5%** | **p-value** |
| --- | --- | --- | --- | --- |
| a (X→M) | 0.96 | 0.89 | 1.03 | <.001 |
| b (M→Y) | 0.21 | 0.18 | 0.24 | <.001 |
| Indirect a×b | 0.2 | 0.17 | 0.23 |  |
| Direct c′ (X→Y\|M) | -0.06 | -0.13 | 0.01 | 0.08 |
| Total c (X→Y) | 0.14 | 0.07 | 0.2 | <.001 |
| a-path PTA moderation (social demand (Within-person)×PTA) | 0.38 | 0.31 | 0.45 | <.001 |

**Table 4.** *Multilevel mediation model testing the indirect effect of subjective auditory demand on fatigue through listening effort, moderated by hearing loss (PTA).*

**Table 4A.** Fixed effects from Models A–C. Model A predicts listening effort from subjective auditory demand (a-path). Model B predicts fatigue from both subjective auditory demand and listening effort (b- and c′-paths). Model C predicts fatigue from subjective auditory demand alone (c-path).

|  |  | **Model a Effort** | | | | | | **Model B Fatigue with Effort** | | | | | | **Model C Fatigue total** | | | | | |
| --- | --- | --- | --- | --- | --- | --- | --- | --- | --- | --- | --- | --- | --- | --- | --- | --- | --- | --- | --- |
|  | **Term** | ***β*** | **SE** | **Lower CI** | **Upper CI** | **t** | **p** | ***β*** | **SE** | **Lower CI** | **Upper CI** | **t** | **p** | ***β*** | **SE** | **Lower CI** | **Upper CI** | **t** | **p** |
| **Subjective Auditory Demand** | Intercept | 5.87 | 1.05 | 3.79 | 7.94 | 5.59 | <.001 | 2.89 | 1.3 | 0.32 | 5.46 | 2.22 | <.05 | 8.28 | 1.46 | 5.4 | 11.17 | 5.68 | <.001 |
|  | Subjective demand (within-person) | 1.81 | 0.04 | 1.73 | 1.89 | 44.64 | <.001 | 0.02 | 0.05 | -0.08 | 0.13 | 0.43 | 0.67 | 0.38 | 0.04 | 0.3 | 0.46 | 8.83 | <.001 |
|  | PTA | 0.67 | 0.12 | 0.43 | 0.92 | 5.47 | <.001 | -0.07 | 0.15 | -0.37 | 0.22 | -0.48 | 0.63 | 0.52 | 0.17 | 0.18 | 0.85 | 3.01 | <.01 |
|  | Subjective demand (between person) | 2.74 | 0.3 | 2.14 | 3.35 | 9 | <.001 | -0.97 | 0.43 | -1.82 | -0.13 | -2.28 | <.05 | 1.42 | 0.42 | 0.58 | 2.25 | 3.34 | <.01 |
|  | Hour | 0.03 | 0.01 | 0.02 | 0.04 | 4.68 | <.001 | 0.12 | 0.01 | 0.11 | 0.14 | 20.62 | <.001 | 0.13 | 0.01 | 0.12 | 0.14 | 21.13 | <.001 |
|  | Sleep quality | -0.35 | 0.11 | -0.57 | -0.14 | -3.31 | <.01 | -0.54 | 0.12 | -0.78 | -0.3 | -4.41 | <.001 | -0.84 | 0.15 | -1.14 | -0.55 | -5.67 | <.001 |
|  | Sleep hours | -0.02 | 0.09 | -0.2 | 0.17 | -0.17 | 0.87 | 0.06 | 0.1 | -0.14 | 0.27 | 0.6 | 0.55 | 0.05 | 0.13 | -0.21 | 0.31 | 0.36 | 0.72 |
|  | Age | -0.03 | 0.01 | -0.05 | -0.01 | -2.53 | <.05 | -0.02 | 0.01 | -0.04 | 0.01 | -1.24 | 0.22 | -0.04 | 0.02 | -0.08 | -0.01 | -2.58 | <.05 |
|  | Sex | -0.01 | 0.23 | -0.46 | 0.44 | -0.05 | 0.96 | -0.62 | 0.25 | -1.11 | -0.13 | -2.49 | <.05 | -0.62 | 0.32 | -1.25 | 0 | -1.97 | 0.05 |
|  | Subjective demand (within-person) × PTA | 0.37 | 0.04 | 0.3 | 0.45 | 9.54 | <.001 | 0 | 0.04 | -0.08 | 0.08 | 0.02 | 0.99 | 0.07 | 0.04 | -0.01 | 0.15 | 1.78 | 0.08 |
|  | Listening effort (within-person) |  |  |  |  |  |  | 0.2 | 0.02 | 0.16 | 0.23 | 11.25 | <.001 |  |  |  |  |  |  |
|  | Listening effort (between-person) |  |  |  |  |  |  | 0.87 | 0.1 | 0.68 | 1.07 | 8.85 | <.001 |  |  |  |  |  |  |

**Table 4B*.*** Summary of indirect, direct, total, and moderated effects. This panel summarizes the path-specific effects derived from Models A–C in Table 3A. The a-path represents the effect of subjective auditory demand on the mediator (listening effort). The b-path represents the effect of the mediator on fatigue, controlling for auditory demand. The direct effect (c′) is the effect of subjective auditory demand on fatigue after accounting for listening effort. The total effect (c) is the effect of social auditory demand on fatigue prior to including the mediator. The indirect effect (a × b) was computed as the product of the within-person a and b coefficients. The final row shows the moderation of the a-path by hearing loss (PTA). Confidence intervals for fixed effects are Wald normal approximations; those for indirect effects are based on Monte Carlo simulations.

| **Effect** | **Estimate** | **2.5%** | **97.5%** | **p-value** |
| --- | --- | --- | --- | --- |
| a (X→M) | 1.81 | 1.73 | 1.89 | <.001 |
| b (M→Y) | 0.2 | 0.16 | 0.23 | <.001 |
| Indirect a×b | 0.35 | 0.29 | 0.42 |  |
| Direct c′ (X→Y\|M) | 0.02 | -0.08 | 0.13 | 0.67 |
| Total c (X→Y) | 0.38 | 0.3 | 0.46 | <.001 |
| a-path PTA moderation (X_wp×PTA) | 0.37 | 0.3 | 0.45 | <.001 |

**Table 5.** *Multilevel mediation model testing the indirect effect of environmental auditory demand on fatigue through affect, moderated by hearing loss (PTA).*

**Table 5A.** Fixed effects from Models A–C. Model A predicts listening effort from environmental auditory demand (a-path). Model B predicts fatigue from both environmental auditory demand and affect (b- and c′-paths). Model C predicts fatigue from environmental auditory demand alone (c-path).

|  |  | **Model a Affect** | | | | | | **Model B Fatigue with Affect** | | | | | | **Model C Fatigue total** | | | | | |
| --- | --- | --- | --- | --- | --- | --- | --- | --- | --- | --- | --- | --- | --- | --- | --- | --- | --- | --- | --- |
|  | **Term** | ***β*** | **SE** | **Lower CI** | **Upper CI** | **t** | **p** | ***β*** | **SE** | **Lower CI** | **Upper CI** | **t** | **p** | ***β*** | **SE** | **Lower CI** | **Upper CI** | **t** | **p** |
| **Environmental Auditory Demand** | Intercept | 2.99 | 1.03 | 0.96 | 5.02 | 2.91 | <.01 | 9.74 | 1.4 | 6.97 | 12.5 | 6.97 | <.001 | 7.65 | 1.51 | 4.66 | 10.64 | 5.07 | <.001 |
|  | Environmental demand (within-person) | 0.03 | 0.02 | -0.01 | 0.06 | 1.61 | 0.11 | 0.08 | 0.02 | 0.04 | 0.13 | 3.81 | <.001 | 0.07 | 0.02 | 0.03 | 0.12 | 3.08 | <.01 |
|  | PTA | -0.14 | 0.11 | -0.35 | 0.07 | -1.35 | 0.18 | 0.69 | 0.14 | 0.41 | 0.97 | 4.92 | <.001 | 0.79 | 0.16 | 0.48 | 1.1 | 5.02 | <.001 |
|  | Environmental demand (between-person) | 0.1 | 0.24 | -0.38 | 0.58 | 0.42 | 0.67 | 0.53 | 0.32 | -0.1 | 1.16 | 1.66 | 0.10 | 0.46 | 0.36 | -0.25 | 1.16 | 1.28 | 0.20 |
|  | Hour | 0.01 | 0 | 0 | 0.02 | 2.96 | <.01 | 0.14 | 0.01 | 0.13 | 0.15 | 23.45 | <.001 | 0.13 | 0.01 | 0.12 | 0.14 | 20.98 | <.001 |
|  | Sleep quality | 0.77 | 0.11 | 0.56 | 0.98 | 7.21 | <.001 | -0.37 | 0.17 | -0.7 | -0.04 | -2.19 | <.05 | -0.89 | 0.16 | -1.2 | -0.57 | -5.63 | <.001 |
|  | Sleep hours | -0.06 | 0.09 | -0.25 | 0.12 | -0.7 | 0.49 | 0.08 | 0.12 | -0.16 | 0.32 | 0.67 | 0.50 | 0.13 | 0.14 | -0.14 | 0.4 | 0.93 | 0.35 |
|  | Age | 0.03 | 0.01 | 0 | 0.05 | 2.21 | <.05 | -0.02 | 0.02 | -0.05 | 0.01 | -1.35 | 0.18 | -0.04 | 0.02 | -0.07 | 0 | -2.21 | <.05 |
|  | Sex | 0 | 0.22 | -0.44 | 0.43 | -0.01 | 0.99 | -0.79 | 0.29 | -1.37 | -0.22 | -2.75 | <.01 | -0.8 | 0.32 | -1.44 | -0.15 | -2.46 | <.05 |
|  | Environmental demand (within-person) × PTA | -0.03 | 0.02 | -0.07 | 0 | -1.72 | 0.09 | 0.06 | 0.02 | 0.02 | 0.1 | 2.75 | <.01 | 0.07 | 0.02 | 0.03 | 0.12 | 3.18 | <.01 |
|  | Affect (within-person) |  |  |  |  |  |  | -0,45 | 0,02 | -0,49 | -0,41 | -22,04 | <.001 |  |  |  |  |  |  |
|  | Affect (between-person) |  |  |  |  |  |  | -0,67 | 0,12 | -0,91 | -0,44 | -5,68 | <.001 |  |  |  |  |  |  |

**Table 5B*.*** Summary of indirect, direct, total, and moderated effects. This panel summarizes the path-specific effects derived from Models A–C in Panel A. The a-path represents the effect of auditory demand on the mediator (affect). The b-path represents the effect of the mediator on fatigue, controlling for auditory demand. The direct effect (c′) is the effect of auditory demand on fatigue after accounting for affect. The total effect (c) is the effect of auditory demand on fatigue prior to including the mediator. The indirect effect (a × b) was computed as the product of the within-person a and b coefficients. The final row shows the moderation of the a-path by hearing loss (PTA). Confidence intervals for fixed effects are Wald normal approximations; those for indirect effects are based on Monte Carlo simulations.

| **Effect** | **Estimate** | **2.5%** | **97.5%** | **p-value** |
| --- | --- | --- | --- | --- |
| a (X→M) | 0.03 | -0.01 | 0.06 | 0.11 |
| b (M→Y) | -0.45 | -0.49 | -0.41 | <.001 |
| Indirect a×b | -0.01 | -0.03 | 0 |  |
| Direct c′ (X→Y\|M) | 0.08 | 0.04 | 0.13 | <.001 |
| Total c (X→Y) | 0.07 | 0.03 | 0.12 | <.01 |
| a-path PTA moderation (environmental auditory demand (within-person) ×PTA) | -0.03 | -0.07 | 0 | 0.09 |

**Table 6.** *Multilevel mediation model testing the indirect effect of social auditory demand on fatigue through affect, moderated by hearing loss (PTA).*

**Table 6A.** Fixed effects from Models A–C. Model A predicts affect from social auditory demand (a-path). Model B predicts fatigue from both social auditory demand and affect (b- and c′-paths). Model C predicts fatigue from social auditory demand alone (c-path).

|  |  | **Model a Affect** | | | | | | **Model B Fatigue with Affect** | | | | | | **Model C Fatigue total** | | | | | |
| --- | --- | --- | --- | --- | --- | --- | --- | --- | --- | --- | --- | --- | --- | --- | --- | --- | --- | --- | --- |
|  | **Term** | ***β*** | **SE** | **Lower CI** | **Upper CI** | **t** | **p** | ***β*** | **SE** | **Lower CI** | **Upper CI** | **t** | **p** | ***β*** | **SE** | **Lower CI** | **Upper CI** | **t** | **p** |
| **Contextual Auditory Demand** | Intercept | 3.01 | 1.03 | 0.97 | 5.05 | 2.92 | <.01 | 9.65 | 1.41 | 6.87 | 12.43 | 6.86 | <.001 | 7.58 | 1.51 | 4.59 | 10.57 | 5.02 | <.001 |
|  | Social demand (within-person) | 0 | 0.03 | -0.05 | 0.06 | 0.15 | 0.88 | 0.13 | 0.03 | 0.07 | 0.2 | 4.21 | <.001 | 0.14 | 0.03 | 0.07 | 0.2 | 3.98 | <.001 |
|  | PTA | -0.15 | 0.11 | -0.36 | 0.06 | -1.37 | 0.17 | 0.7 | 0.14 | 0.42 | 0.98 | 4.99 | <.001 | 0.8 | 0.16 | 0.49 | 1.11 | 5.1 | <.001 |
|  | Social demand (person mean-centered) | -0.13 | 0.3 | -0.71 | 0.46 | -0.42 | 0.67 | 0.51 | 0.39 | -0.27 | 1.28 | 1.3 | 0.20 | 0.59 | 0.43 | -0.27 | 1.45 | 1.36 | 0.18 |
|  | Hour | 0.01 | 0 | 0.01 | 0.02 | 3.03 | <.01 | 0.14 | 0.01 | 0.13 | 0.15 | 23.86 | <.001 | 0.13 | 0.01 | 0.12 | 0.14 | 21.31 | <.001 |
|  | Sleep quality | 0.79 | 0.11 | 0.58 | 1 | 7.4 | <.001 | -0.36 | 0.17 | -0.7 | -0.03 | -2.15 | <.05 | -0.88 | 0.16 | -1.19 | -0.57 | -5.64 | <.001 |
|  | Sleep hours | -0.07 | 0.09 | -0.25 | 0.11 | -0.74 | 0.46 | 0.07 | 0.12 | -0.17 | 0.32 | 0.61 | 0.54 | 0.12 | 0.14 | -0.15 | 0.39 | 0.9 | 0.37 |
|  | Age | 0.03 | 0.01 | 0 | 0.05 | 2.09 | <.05 | -0.02 | 0.02 | -0.05 | 0.01 | -1.37 | 0.17 | -0.04 | 0.02 | -0.07 | 0 | -2.17 | <.05 |
|  | Sex | -0.02 | 0.22 | -0.46 | 0.43 | -0.07 | 0.95 | -0.75 | 0.29 | -1.33 | -0.17 | -2.57 | <.05 | -0.75 | 0.33 | -1.39 | -0.1 | -2.28 | <.05 |
|  | Social demand (within-person) × PTA | -0.02 | 0.03 | -0.07 | 0.03 | -0.74 | 0.46 | 0.08 | 0.03 | 0.02 | 0.14 | 2.65 | <.01 | 0.09 | 0.03 | 0.03 | 0.16 | 2.78 | <.01 |
|  | Affect (within-person) |  |  |  |  |  |  | -0.45 | 0.02 | -0.49 | -0.41 | -22.12 | <.001 |  |  |  |  |  |  |
|  | Affect (person-mean centered) |  |  |  |  |  |  | -0.66 | 0.12 | -0.89 | -0.42 | -5.54 | <.001 |  |  |  |  |  |  |

**Table 6B*.*** Summary of indirect, direct, total, and moderated effects. This panel summarizes the path-specific effects derived from Models A–C in Panel A. The a-path represents the effect of auditory demand on the mediator (affect). The b-path represents the effect of the mediator on fatigue, controlling for auditory demand. The direct effect (c′) is the effect of auditory demand on fatigue after accounting for affect. The total effect (c) is the effect of auditory demand on fatigue prior to including the mediator. The indirect effect (a × b) was computed as the product of the within-person a and b coefficients. The final row shows the moderation of the a-path by hearing loss (PTA). Confidence intervals for fixed effects are Wald normal approximations; those for indirect effects are based on Monte Carlo simulations.

| **Effect** | **Estimate** | **2.5%** | **97.5%** | **p-value** |
| --- | --- | --- | --- | --- |
| a (X→M) | 0 | -0.05 | 0.06 | 0.88 |
| b (M→Y) | -0.45 | -0.49 | -0.41 | <.001 |
| Indirect a×b | 0 | -0.03 | 0.02 |  |
| Direct c′ (X→Y\|M) | 0.13 | 0.07 | 0.2 | <.001 |
| Total c (X→Y) | 0.14 | 0.07 | 0.2 | <.001 |
| a-path PTA moderation (social demand (Within-person)×PTA) | -0.02 | -0.07 | 0.03 | 0.46 |

**Table 7.** *Multilevel mediation model testing the indirect effect of subjective auditory demand on fatigue through affect, moderated by hearing loss (PTA).*

**Table 7A.** Fixed effects from Models A–C. Model A predicts affect from subjective auditory demand (a-path). Model B predicts fatigue from both subjective auditory demand and affect (b- and c′-paths). Model C predicts fatigue from subjective auditory demand alone (c-path).

|  |  | **Model a Affect** | | | | | | **Model B Fatigue with Affect** | | | | | | **Model C Fatigue total** | | | | | |
| --- | --- | --- | --- | --- | --- | --- | --- | --- | --- | --- | --- | --- | --- | --- | --- | --- | --- | --- | --- |
|  | **Term** | ***β*** | **SE** | **Lower CI** | **Upper CI** | **t** | **p** | ***β*** | **SE** | **Lower CI** | **Upper CI** | **t** | **p** | ***β*** | **SE** | **Lower CI** | **Upper CI** | **t** | **p** |
| **Subjective Auditory Demand** | Intercept | 2.8 | 1.02 | 0.79 | 4.82 | 2.75 | <.01 | 10.09 | 1.37 | 7.38 | 12.79 | 7.38 | <.001 | 8.28 | 1.46 | 5.4 | 11.17 | 5.68 | <.001 |
|  | Social demand (within-person) | -0.1 | 0.03 | -0.17 | -0.04 | -3.05 | <.01 | 0.33 | 0.04 | 0.25 | 0.41 | 8.22 | <.001 | 0.38 | 0.04 | 0.3 | 0.46 | 8.83 | <.001 |
|  | PTA | -0.05 | 0.12 | -0.29 | 0.19 | -0.43 | 0.67 | 0.49 | 0.16 | 0.18 | 0.79 | 3.13 | <.01 | 0.52 | 0.17 | 0.18 | 0.85 | 3.01 | <.01 |
|  | Subjective demand (person mean-centered) | -0.47 | 0.3 | -1.05 | 0.12 | -1.58 | 0.12 | 1.12 | 0.39 | 0.36 | 1.89 | 2.9 | <.01 | 1.42 | 0.42 | 0.58 | 2.25 | 3.34 | <.01 |
|  | Hour | 0.02 | 0 | 0.01 | 0.02 | 3.24 | <.01 | 0.14 | 0.01 | 0.13 | 0.15 | 23.64 | <.001 | 0.13 | 0.01 | 0.12 | 0.14 | 21.13 | <.001 |
|  | Sleep quality | 0.78 | 0.1 | 0.57 | 0.98 | 7.49 | <.001 | -0.36 | 0.16 | -0.69 | -0.04 | -2.24 | <.05 | -0.84 | 0.15 | -1.14 | -0.55 | -5.67 | <.001 |
|  | Sleep hours | -0.05 | 0.09 | -0.23 | 0.14 | -0.5 | 0.62 | 0.02 | 0.12 | -0.22 | 0.25 | 0.14 | 0.89 | 0.05 | 0.13 | -0.21 | 0.31 | 0.36 | 0.72 |
|  | Age | 0.03 | 0.01 | 0 | 0.05 | 2.26 | <.05 | -0.03 | 0.02 | -0.06 | 0 | -1.76 | 0.08 | -0.04 | 0.02 | -0.08 | -0.01 | -2.58 | <.05 |
|  | Sex | -0.07 | 0.22 | -0.5 | 0.37 | -0.3 | 0.77 | -0.66 | 0.29 | -1.23 | -0.09 | -2.31 | <.05 | -0.62 | 0.32 | -1.25 | 0 | -1.97 | 0.05 |
|  | Subjective demand (within-person) × PTA | -0.04 | 0.03 | -0.11 | 0.02 | -1.37 | 0.17 | 0.05 | 0.04 | -0.02 | 0.13 | 1.36 | 0.17 | 0.07 | 0.04 | -0.01 | 0.15 | 1.78 | 0.08 |
|  | Affect (within-person) |  |  |  |  |  |  | -0.44 | 0.02 | -0.48 | -0.4 | -21.62 | <.001 |  |  |  |  |  |  |
|  | Affect (person-mean centered) |  |  |  |  |  |  | -0.62 | 0.12 | -0.85 | -0.38 | -5.27 | <.001 |  |  |  |  |  |  |

**Table 7B*.*** Summary of indirect, direct, total, and moderated effects. This panel summarizes the path-specific effects derived from Models A–C in Panel A. The a-path represents the effect of auditory demand on the mediator (affect). The b-path represents the effect of the mediator on fatigue, controlling for auditory demand. The direct effect (c′) is the effect of auditory demand on fatigue after accounting for affect. The total effect (c) is the effect of auditory demand on fatigue prior to including the mediator. The indirect effect (a × b) was computed as the product of the within-person a and b coefficients. The final row shows the moderation of the a-path by hearing loss (PTA). Confidence intervals for fixed effects are Wald normal approximations; those for indirect effects are based on Monte Carlo simulations.

| **Effect** | **Estimate** | **2.5%** | **97.5%** | **p-value** |
| --- | --- | --- | --- | --- |
| a (X→M) | -0.1 | -0.17 | -0.04 | <.01 |
| b (M→Y) | -0.44 | -0.48 | -0.4 | <.001 |
| Indirect a×b | 0.05 | 0.02 | 0.07 |  |
| Direct c′ (X→Y\|M) | 0.33 | 0.25 | 0.41 | <.001 |
| Total c (X→Y) | 0.38 | 0.3 | 0.46 | <.001 |
| a-path PTA moderation (subjective demand(within-person) × PTA) | -0.04 | -0.11 | 0.02 | 0.17 |

**Table 8.** Distribution of auditory demand per participant.

|  |  | **Environmental** | | | | **Social** | | | **Subjective** | | |
| --- | --- | --- | --- | --- | --- | --- | --- | --- | --- | --- | --- |
| **Subject** | **N prompts** | **Least (0) %** | **Little (1) %** | **Some (2) %** | **Most (3) %** | **Least (0)%** | **Some (1)%** | **Most (2)%** | **Least(0)%** | **Some (1) %** | **Most (2) %** |
| 1 | 32 | 18.8 | 43.8 | 21.9 | 15.6 | 15.6 | 56.2 | 28.1 | 87.5 | 12.5 | 0 |
| 2 | 8 | 25 | 12.5 | 25 | 37.5 | 25 | 62.5 | 12.5 | 50 | 50 | 0 |
| 3 | 24 | 37.5 | 20.8 | 16.7 | 25 | 33.3 | 41.7 | 25 | 70.8 | 29.2 | 0 |
| 4 | 15 | 6.7 | 6.7 | 60 | 26.7 | 18.8 | 37.5 | 43.8 | 6.7 | 40 | 53.3 |
| 5 | 22 | 27.3 | 22.7 | 27.3 | 22.7 | 54.5 | 22.7 | 22.7 | 72.7 | 27.3 | 0 |
| 6 | 35 | 5.7 | 5.7 | 48.6 | 40 | 17.1 | 31.4 | 51.4 | 22.9 | 65.7 | 11.4 |
| 7 | 30 | 43.3 | 16.7 | 36.7 | 3.3 | 26.7 | 56.7 | 16.7 | 70 | 20 | 10 |
| 8 | 27 | 7.4 | 33.3 | 40.7 | 18.5 | 40.7 | 48.1 | 11.1 | 63 | 33.3 | 3.7 |
| 9 | 24 | 12.5 | 25 | 45.8 | 16.7 | 12.5 | 62.5 | 25 | 70.8 | 29.2 | 0 |
| 10 | 26 | 42.3 | 23.1 | 26.9 | 7.7 | 46.2 | 46.2 | 7.7 | 92 | 8 | 0 |
| 11 | 26 | 19.2 | 38.5 | 38.5 | 3.8 | 65.4 | 30.8 | 3.8 | 57.7 | 42.3 | 0 |
| 12 | 32 | 6.2 | 25 | 37.5 | 31.2 | 25 | 25 | 50 | 31.2 | 56.2 | 12.5 |
| 13 | 7 | 57.1 | 0 | 28.6 | 14.3 | 57.1 | 42.9 | 0 | 71.4 | 14.3 | 14.3 |
| 14 | 26 | 34.6 | 3.8 | 42.3 | 19.2 | 38.5 | 38.5 | 23.1 | 100 | 0 | 0 |
| 15 | 22 | 59.1 | 36.4 | 0 | 4.5 | 95.5 | 0 | 4.5 | 86.4 | 13.6 | 0 |
| 16 | 28 | 14.3 | 25 | 53.6 | 7.1 | 7.1 | 0 | 92.9 | 92.9 | 7.1 | 0 |
| 17 | 32 | 21.9 | 18.8 | 34.4 | 25 | 15.6 | 56.2 | 28.1 | 31.2 | 53.1 | 15.6 |
| 18 | 16 | 25 | 0 | 18.8 | 56.2 | 25 | 31.2 | 43.8 | 31.2 | 37.5 | 31.2 |
| 19 | 39 | 15.4 | 7.7 | 41 | 35.9 | 17.9 | 17.9 | 64.1 | 30.8 | 38.5 | 30.8 |
| 20 | 13 | 15.4 | 15.4 | 15.4 | 53.8 | 7.7 | 0 | 92.3 | 69.2 | 30.8 | 0 |
| 21 | 38 | 0 | 0 | 31.6 | 68.4 | 0 | 34.2 | 65.8 | 28.9 | 57.9 | 13.2 |
| 22 | 28 | 25 | 28.6 | 35.7 | 10.7 | 32.1 | 53.6 | 14.3 | 71.4 | 28.6 | 0 |
| 23 | 38 | 42.1 | 13.2 | 26.3 | 18.4 | 65.8 | 2.6 | 31.6 | 50 | 39.5 | 10.5 |
| 24 | 22 | 22.7 | 27.3 | 36.4 | 13.6 | 36.4 | 45.5 | 18.2 | 40.9 | 40.9 | 18.2 |
| 25 | 38 | 21.1 | 13.2 | 36.8 | 28.9 | 26.3 | 57.9 | 15.8 | 44.7 | 44.7 | 10.5 |
| 26 | 34 | 38.2 | 2.9 | 35.3 | 23.5 | 38.2 | 58.8 | 2.9 | 23.5 | 41.2 | 35.3 |
| 27 | 19 | 0 | 0 | 73.7 | 26.3 | 0 | 94.7 | 5.3 | 0 | 94.7 | 5.3 |
| 28 | 36 | 36.1 | 27.8 | 19.4 | 16.7 | 22.2 | 69.4 | 8.3 | 0 | 100 | 0 |
| 29 | 30 | 16.7 | 40 | 13.3 | 30 | 48.4 | 38.7 | 12.9 | 10 | 90 | 0 |
| 30 | 34 | 17.6 | 8.8 | 38.2 | 35.3 | 32.4 | 26.5 | 41.2 | 44.1 | 38.2 | 17.6 |
| 31 | 16 | 25 | 31.2 | 18.8 | 25 | 18.8 | 43.8 | 37.5 | 6.2 | 93.8 | 0 |
| 32 | 23 | 43.5 | 4.3 | 30.4 | 21.7 | 39.1 | 43.5 | 17.4 | 50 | 27.3 | 22.7 |
| 33 | 28 | 53.6 | 10.7 | 25 | 10.7 | 64.3 | 0 | 35.7 | 42.9 | 50 | 7.1 |
| 34 | 32 | 37.5 | 46.9 | 15.6 | 0 | 78.1 | 0 | 21.9 | 53.1 | 43.8 | 3.1 |
| 35 | 31 | 32.3 | 19.4 | 25.8 | 22.6 | 41.9 | 35.5 | 22.6 | 41.9 | 54.8 | 3.2 |
| 36 | 5 | 20 | 60 | 20 | 0 | 0 | 60 | 40 | 0 | 60 | 40 |
| 37 | 38 | 26.3 | 31.6 | 31.6 | 10.5 | 36.8 | 55.3 | 7.9 | 57.9 | 42.1 | 0 |
| 38 | 35 | 14.3 | 11.4 | 48.6 | 25.7 | 5.7 | 54.3 | 40 | 45.7 | 54.3 | 0 |
| 39 | 30 | 10 | 3.3 | 60 | 26.7 | 13.3 | 56.7 | 30 | 60 | 30 | 10 |
| 40 | 33 | 6.1 | 15.2 | 60.6 | 18.2 | 6.1 | 69.7 | 24.2 | 12.1 | 84.8 | 3 |
| 41 | 32 | 31.2 | 9.4 | 21.9 | 37.5 | 25 | 37.5 | 37.5 | 65.6 | 21.9 | 12.5 |
| 42 | 37 | 45.9 | 10.8 | 27 | 16.2 | 45.9 | 29.7 | 24.3 | 35.1 | 59.5 | 5.4 |
| 43 | 34 | 32.4 | 35.3 | 20.6 | 11.8 | 23.5 | 35.3 | 41.2 | 2.9 | 47.1 | 50 |
| 44 | 24 | 12.5 | 12.5 | 54.2 | 20.8 | 26.9 | 30.8 | 42.3 | 33.3 | 66.7 | 0 |
| 45 | 32 | 53.1 | 12.5 | 28.1 | 6.2 | 78.1 | 0 | 21.9 | 59.4 | 12.5 | 28.1 |
| 46 | 32 | 12.5 | 21.9 | 59.4 | 6.2 | 25 | 68.8 | 6.2 | 50 | 50 | 0 |
| 47 | 28 | 46.4 | 17.9 | 25 | 10.7 | 32.1 | 60.7 | 7.1 | 60.7 | 35.7 | 3.6 |
| 48 | 40 | 7.5 | 5 | 57.5 | 30 | 5 | 60 | 35 | 5 | 87.5 | 7.5 |
| 49 | 35 | 17.1 | 2.9 | 25.7 | 54.3 | 20 | 20 | 60 | 28.6 | 62.9 | 8.6 |
| 50 | 32 | 9.4 | 3.1 | 12.5 | 75 | 12.5 | 53.1 | 34.4 | 93.8 | 6.2 | 0 |
| 51 | 15 | 46.7 | 0 | 33.3 | 20 | 40 | 0 | 60 | 60 | 20 | 20 |
| 52 | 33 | 33.3 | 9.1 | 45.5 | 12.1 | 15.2 | 15.2 | 69.7 | 42.4 | 36.4 | 21.2 |
| 53 | 34 | 20.6 | 20.6 | 35.3 | 23.5 | 41.2 | 32.4 | 26.5 | 64.7 | 35.3 | 0 |
| 54 | 22 | 63.6 | 4.5 | 13.6 | 18.2 | 68.2 | 4.5 | 27.3 | 85.7 | 14.3 | 0 |
| 55 | 28 | 10.7 | 0 | 35.7 | 53.6 | 3.6 | 32.1 | 64.3 | 3.6 | 25 | 71.4 |
| 56 | 36 | 11.1 | 2.8 | 72.2 | 13.9 | 5.6 | 75 | 19.4 | 83.3 | 16.7 | 0 |
| 57 | 37 | 21.6 | 16.2 | 54.1 | 8.1 | 5.4 | 78.4 | 16.2 | 81.1 | 18.9 | 0 |
| 58 | 22 | 40.9 | 13.6 | 40.9 | 4.5 | 22.7 | 63.6 | 13.6 | 9.1 | 81.8 | 9.1 |
| 59 | 18 | 0 | 100 | 0 | 0 | 100 | 0 | 0 | 0 | 94.4 | 5.6 |
| 60 | 33 | 15.2 | 9.1 | 54.5 | 21.2 | 12.1 | 33.3 | 54.5 | 78.1 | 21.9 | 0 |
| 61 | 34 | 44.1 | 17.6 | 26.5 | 11.8 | 47.1 | 23.5 | 29.4 | 73.5 | 23.5 | 2.9 |
| 62 | 31 | 32.3 | 3.2 | 58.1 | 6.5 | 25 | 43.8 | 31.2 | 28.1 | 43.8 | 28.1 |
| 63 | 29 | 34.5 | 10.3 | 20.7 | 34.5 | 24.1 | 34.5 | 41.4 | 31 | 13.8 | 55.2 |
| 64 | 6 | 66.7 | 16.7 | 16.7 | 0 | 83.3 | 0 | 16.7 | 83.3 | 16.7 | 0 |
| 65 | 39 | 61.5 | 30.8 | 5.1 | 2.6 | 82.1 | 0 | 17.9 | 92.3 | 7.7 | 0 |
| 66 | 17 | 41.2 | 11.8 | 23.5 | 23.5 | 64.7 | 11.8 | 23.5 | 82.4 | 11.8 | 5.9 |
| 67 | 16 | 12.5 | 12.5 | 50 | 25 | 18.8 | 31.2 | 50 | 56.2 | 43.8 | 0 |
| 68 | 33 | 21.2 | 18.2 | 30.3 | 30.3 | 9.1 | 42.4 | 48.5 | 39.4 | 48.5 | 12.1 |
| 69 | 21 | 42.9 | 14.3 | 23.8 | 19 | 38.1 | 0 | 61.9 | 33.3 | 52.4 | 14.3 |
| 70 | 36 | 66.7 | 5.6 | 16.7 | 11.1 | 55.6 | 27.8 | 16.7 | 61.1 | 36.1 | 2.8 |
| 71 | 31 | 32.3 | 19.4 | 38.7 | 9.7 | 32.3 | 51.6 | 16.1 | 80.6 | 19.4 | 0 |
| 72 | 32 | 43.8 | 0 | 37.5 | 18.8 | 43.8 | 53.1 | 3.1 | 53.1 | 31.2 | 15.6 |
| 73 | 38 | 47.4 | 7.9 | 26.3 | 18.4 | 44.7 | 31.6 | 23.7 | 47.4 | 52.6 | 0 |
| 74 | 11 | 36.4 | 9.1 | 9.1 | 45.5 | 45.5 | 0 | 54.5 | 18.2 | 72.7 | 9.1 |
| 75 | 39 | 46.2 | 20.5 | 28.2 | 5.1 | 51.3 | 41 | 7.7 | 74.4 | 25.6 | 0 |
| 76 | 39 | 41 | 5.1 | 25.6 | 28.2 | 20.5 | 69.2 | 10.3 | 51.3 | 43.6 | 5.1 |
| 77 | 13 | 7.7 | 30.8 | 46.2 | 15.4 | 30.8 | 0 | 69.2 | 69.2 | 23.1 | 7.7 |
| 78 | 37 | 29.7 | 27 | 32.4 | 10.8 | 43.2 | 0 | 56.8 | 43.2 | 35.1 | 21.6 |
| 79 | 16 | 18.8 | 12.5 | 25 | 43.8 | 31.2 | 43.8 | 25 | 62.5 | 18.8 | 18.8 |
| 80 | 48 | 37.5 | 14.6 | 39.6 | 8.3 | 52.1 | 16.7 | 31.2 | 58.3 | 39.6 | 2.1 |
| 81 | 35 | 11.4 | 11.4 | 60 | 17.1 | 31.4 | 45.7 | 22.9 | 68.6 | 20 | 11.4 |
| 82 | 40 | 0 | 5 | 80 | 15 | 0 | 77.5 | 22.5 | 97.5 | 2.5 | 0 |
| 83 | 45 | 40 | 4.4 | 44.4 | 11.1 | 13.3 | 75.6 | 11.1 | 62.2 | 22.2 | 15.6 |
| 84 | 26 | 7.7 | 19.2 | 50 | 23.1 | 57.7 | 11.5 | 30.8 | 61.5 | 38.5 | 0 |
| 85 | 36 | 30.6 | 19.4 | 16.7 | 33.3 | 52.8 | 0 | 47.2 | 55.6 | 38.9 | 5.6 |
| 86 | 34 | 52.9 | 0 | 23.5 | 23.5 | 44.1 | 32.4 | 23.5 | 52.9 | 32.4 | 14.7 |
| 87 | 31 | 16.1 | 19.4 | 25.8 | 38.7 | 12.9 | 38.7 | 48.4 | 6.5 | 38.7 | 54.8 |
| 88 | 28 | 39.3 | 3.6 | 28.6 | 28.6 | 25 | 32.1 | 42.9 | 85.7 | 14.3 | 0 |
| 89 | 27 | 25.9 | 37 | 18.5 | 18.5 | 74.1 | 7.4 | 18.5 | 96.3 | 3.7 | 0 |
| 90 | 39 | 35.9 | 38.5 | 15.4 | 10.3 | 61.5 | 20.5 | 17.9 | 71.8 | 28.2 | 0 |
| 91 | 38 | 21.1 | 5.3 | 44.7 | 28.9 | 18.4 | 50 | 31.6 | 22.2 | 55.6 | 22.2 |
| 92 | 39 | 33.3 | 25.6 | 15.4 | 25.6 | 48.7 | 35.9 | 15.4 | 56.4 | 35.9 | 7.7 |
| 93 | 37 | 35.1 | 8.1 | 27 | 29.7 | 24.3 | 45.9 | 29.7 | 37.8 | 48.6 | 13.5 |
| 94 | 28 | 25 | 17.9 | 39.3 | 17.9 | 28.6 | 39.3 | 32.1 | 48.1 | 37 | 14.8 |
| 95 | 20 | 10 | 0 | 75 | 15 | 25 | 75 | 0 | 5 | 10 | 85 |
| 96 | 27 | 11.1 | 14.8 | 14.8 | 59.3 | 14.8 | 0 | 85.2 | 3.7 | 33.3 | 63 |
| 97 | 35 | 31.4 | 14.3 | 42.9 | 11.4 | 40 | 31.4 | 28.6 | 40 | 31.4 | 28.6 |
| 98 | 35 | 37.1 | 28.6 | 8.6 | 25.7 | 20 | 51.4 | 28.6 | 28.6 | 45.7 | 25.7 |
| 99 | 20 | 45 | 25 | 15 | 15 | 75 | 10 | 15 | 55 | 35 | 10 |
| 100 | 35 | 25.7 | 8.6 | 37.1 | 28.6 | 31.4 | 42.9 | 25.7 | 17.1 | 68.6 | 14.3 |
| 101 | 29 | 27.6 | 0 | 55.2 | 17.2 | 30 | 50 | 20 | 62.1 | 34.5 | 3.4 |
| 102 | 33 | 48.5 | 30.3 | 18.2 | 3 | 88.2 | 0 | 11.8 | 79.4 | 20.6 | 0 |
| 103 | 36 | 25 | 16.7 | 25 | 33.3 | 19.4 | 36.1 | 44.4 | 55.6 | 38.9 | 5.6 |
| 104 | 27 | 25.9 | 14.8 | 14.8 | 44.4 | 25.9 | 55.6 | 18.5 | 66.7 | 29.6 | 3.7 |
| 105 | 26 | 11.5 | 26.9 | 19.2 | 42.3 | 19.2 | 34.6 | 46.2 | 15.4 | 26.9 | 57.7 |
| 106 | 29 | 24.1 | 44.8 | 13.8 | 17.2 | 3.4 | 48.3 | 48.3 | 93.1 | 6.9 | 0 |
| 107 | 38 | 28.9 | 21.1 | 21.1 | 28.9 | 26.3 | 47.4 | 26.3 | 44.7 | 42.1 | 13.2 |
| 108 | 33 | 36.4 | 3 | 42.4 | 18.2 | 30.3 | 39.4 | 30.3 | 36.4 | 33.3 | 30.3 |
| 109 | 36 | 22.2 | 2.8 | 33.3 | 41.7 | 5.6 | 52.8 | 41.7 | 75 | 22.2 | 2.8 |
| 110 | 22 | 40.9 | 13.6 | 22.7 | 22.7 | 18.2 | 68.2 | 13.6 | 36.4 | 40.9 | 22.7 |
| 111 | 27 | 40.7 | 7.4 | 29.6 | 22.2 | 48.1 | 37 | 14.8 | 40.7 | 37 | 22.2 |
| 112 | 34 | 14.7 | 32.4 | 20.6 | 32.4 | 32.4 | 47.1 | 20.6 | 14.7 | 82.4 | 2.9 |
| 113 | 15 | 20 | 20 | 13.3 | 46.7 | 33.3 | 40 | 26.7 | 57.1 | 14.3 | 28.6 |
| 114 | 32 | 28.1 | 9.4 | 40.6 | 21.9 | 43.8 | 0 | 56.2 | 28.1 | 43.8 | 28.1 |
| 115 | 31 | 32.3 | 16.1 | 35.5 | 16.1 | 38.7 | 48.4 | 12.9 | 96.8 | 3.2 | 0 |
| 116 | 30 | 53.3 | 20 | 13.3 | 13.3 | 6.7 | 46.7 | 46.7 | 56.7 | 43.3 | 0 |
| 117 | 33 | 27.3 | 15.2 | 18.2 | 39.4 | 27.3 | 21.2 | 51.5 | 0 | 45.5 | 54.5 |
| 118 | 29 | 55.2 | 10.3 | 20.7 | 13.8 | 58.6 | 24.1 | 17.2 | 89.7 | 10.3 | 0 |
| 119 | 28 | 32.1 | 7.1 | 42.9 | 17.9 | 17.9 | 28.6 | 53.6 | 32.1 | 64.3 | 3.6 |
| 120 | 36 | 30.6 | 2.8 | 41.7 | 25 | 36.1 | 25 | 38.9 | 30.6 | 66.7 | 2.8 |
| 121 | 22 | 22.7 | 4.5 | 72.7 | 0 | 27.3 | 50 | 22.7 | 18.2 | 54.5 | 27.3 |
| 122 | 29 | 17.2 | 10.3 | 44.8 | 27.6 | 27.6 | 44.8 | 27.6 | 93.1 | 6.9 | 0 |
| 123 | 27 | 40.7 | 7.4 | 22.2 | 29.6 | 48.1 | 14.8 | 37 | 63 | 22.2 | 14.8 |
| 124 | 17 | 41.2 | 17.6 | 23.5 | 17.6 | 41.2 | 52.9 | 5.9 | 58.8 | 29.4 | 11.8 |
| 125 | 31 | 16.1 | 9.7 | 45.2 | 29 | 25.8 | 22.6 | 51.6 | 25.8 | 38.7 | 35.5 |
| 126 | 29 | 13.8 | 10.3 | 58.6 | 17.2 | 20.7 | 62.1 | 17.2 | 75.9 | 20.7 | 3.4 |
| 127 | 27 | 22.2 | 7.4 | 44.4 | 25.9 | 11.1 | 63 | 25.9 | 33.3 | 37 | 29.6 |
| 128 | 32 | 50 | 15.6 | 18.8 | 15.6 | 68.8 | 6.2 | 25 | 65.6 | 25 | 9.4 |
| 129 | 40 | 10 | 5 | 60 | 25 | 5 | 50 | 45 | 70 | 25 | 5 |
| 130 | 25 | 24 | 24 | 20 | 32 | 12 | 0 | 88 | 48 | 32 | 20 |
